# Systemic Treatments and Molecular Biomarkers for Perivascular Epithelioid Cell Tumors: A Single-institution Retrospective Analysis

**DOI:** 10.1158/2767-9764.CRC-23-0139

**Published:** 2023-07-12

**Authors:** Stefano Testa, Nam Q. Bui, Kristen N. Ganjoo

**Affiliations:** 1 Department of Medicine, Stanford University, Stanford, California.; 2 Division of Oncology, Department of Medicine, Stanford University, Stanford, California.

## Abstract

Perivascular epithelioid cell tumors (PEComa) are a large family of mesenchymal neoplasms, with variable clinical course. Evidence regarding treatment of advanced PEComas is scarce, with only one FDA-approved treatment available. The goals of this study were to provide data regarding systemic treatments for advanced PEComas and to identify biomarkers of prognostic relevance. This is a single-institution retrospective study of patients with advanced PEComas requiring systemic treatment, including malignant PEComa, angiomyolipoma (including the epithelioid variant), and lymphangioleiomyomatosis. Outcomes measured were overall survival (OS), first-line and combined progression-free survival (PFS), and tumor response. Kaplan–Meier, univariable, and multivariable Cox proportional hazards analysis were performed. A total of 29 patients were included, most with malignant PEComa (*n* = 17). Median OS was 204.9 months, while median PFS was 92.4 months from first-line, and 15.8 months for all lines combined. TFE3 overexpression correlated with higher risk of death (HR: 11.8, *P* = 0.04), and shorter median OS (*P* = 0.001). Chemotherapy and mTOR inhibitors showed similar OS (*P* = 0.84), and first-line PFS (*P* = 0.67). Combined PFS was similar between individual mTOR inhibitors, chemotherapy, immune checkpoint inhibitors and other treatments (*P* = 0.19). Different mTOR inhibitors demonstrated similar efficacy, making cost and availability important considerations when choosing a specific agent. mTOR inhibitors showed similar outcomes as chemotherapy, suggesting that these should be preferred whenever possible for patients with PEComas given the morbidity associated with chemotherapy. TFE3 overexpression highlighted a subgroup of PEComas with worse prognosis and more aggressive behavior.

**Significance:**

This study examines systemic treatments for advanced PEComas, a rare group of sarcomas, and identifies molecular biomarkers of prognosis. Our results show that mTOR inhibitors have similar efficacy as chemotherapy, and that TFE3 overexpression, on IHC or FISH, correlates with a more aggressive disease course.

## Introduction

Perivascular epithelioid cell tumors (PEComa) are a heterogeneous family of soft-tissue sarcomas (STS) characterized by the presence of epithelioid cells with perivascular distribution and a peculiar IHC profile with strong expression of melanocytic and smooth muscle markers (1–3).

Several tumors belong to the PEComa family, including angiomyolipomas (AML), lymphangioleiomyomatosis (LAM), malignant PEComas, epithelioid AML, and clear-cell sugar tumor of the lung (1). The clinical behavior and biology of PEComas varies among the different histotypes. For example, AML and LAM most often show a benign indolent course with low to no risk of developing metastases, and can be almost always treated with surgery alone, while other histotypes like malignant PEComas and epithelioid AML have a more aggressive behavior showing local invasion and increased metastatic potential, often requiring systemic therapy. The incidence of the different types of PEComas also varies, with malignant PEComas being classified as an ultra-rare STS with annual incidence of 1/1,000,000 and AML showing a prevalence of up to 2.2% in asymptomatic adults undergoing imaging for other reasons (4, 5). PEComas arise more frequently in females and can originate virtually in any body location, with a predilection for visceral sites including the kidney, lungs, retroperitoneum, and the uterus.

The pathogenesis of PEComas is linked to constitutive activation of the mTOR pathway (6). PEComas often harbor somatic inactivating mutations in either *TSC2* or *TSC1*, and patients with tuberous sclerosis complex, who carry germline mutations in these genes, often develop various types of PEComas during their lifetime (7). The *TSC2* and *TSC1* gene products negatively regulate the mTOR complex 1 preventing constitutive activation of the mTOR pathway (8). On the basis of this observation, mTOR inhibitors including sirolimus, everolimus, and temsirolimus, have been used in the treatment of PEComas with good clinical results as reported mostly in retrospective reviews and case reports (9–15). In addition, the only FDA-approved treatment available for advanced malignant PEComa in adults is nab-sirolimus, a parenteral nanoparticle albumin-bound version of sirolimus, which has been approved on the basis of the results of the AMPECT trial (16). Other treatment modalities, including cytotoxic chemotherapy, tyrosine kinase inhibitors, and antiangiogenic agents, have also been used for patients with PEComas, but are overall less studied and not as effective as mTOR inhibitors (refs. 17–19; Supplementary Table S1).

Here we report on the clinical outcomes of patients with PEComas treated at our institution over the last 22 years, focusing on those with advanced disease that required systemic treatment. Specifically, we compared outcomes of patients with PEComas that received different systemic treatment modalities and explored the prognostic and predictive role of several patient characteristics and tumor biomarkers by correlating these with outcomes. In addition, we reviewed the current literature on systemic treatment of PEComas and compared it with our findings, hoping to provide valuable insights into the effectiveness of different systemic therapies for this rare and poorly understood tumor family that can help guide clinical management.

## Materials and Methods

### Patient Selection

Patients were selected from a database of pathology cases of PEComa, AML (including the epithelioid variant), and LAM reviewed at the Stanford Pathology Department (Supplementary Fig. S1) between 2000 and 2022. A pathologic diagnosis of malignant PEComa was made if more than two of the following features were present: size > 5 cm, infiltrative growth pattern, high-grade nuclear atypia, high cellularity, mitoses > 1 per 50 high-power field, necrosis or lymphovascular invasion (20). Patients that required systemic treatment, either because of presence of metastases, unresectable disease, or for symptomatic control were included in the analysis. Patients treated with surgery only, those that received their treatment entirely at an outside institution, and those diagnosed incidentally during another surgery were excluded from the analysis. Patients with LAM that underwent lung transplant before receiving mTOR inhibitors or other systemic therapies were excluded as well. There were no inclusion limitations in terms of age or primary tumor site.

### Data Collection

Patient information was retrospectively collected through review of patients’ charts and physician documentation. Data collected included demographics, treatment regimens, clinical outcomes, radiographic response, and next-generation sequencing (NGS) data. NGS was available for 12 of 29 patients and obtained through either the Stanford Actionable Mutation Panel for Solid Tumors (STAMP) panel (*n* = 2; refs. 21, 22), the Foundation One panel (Foundation Medicine, *n* = 4), the Altera Tumor Genomics Profiling panel (Natera, *n* = 2), the UCSF 500 Cancer Gene panel (*n* = 2), or the GPS cancer test (NantHealth, *n* = 1). One patient had a germline *TSC2* mutation detected with the NGS panel from PreventionGenetics.

Of the 59 treatment episodes recorded, 40 had available radiographic scans (CT scans or MRI) that allowed direct measurements of the change in maximum tumor diameters pretreatment and posttreatment for assessment of response based on RECIST 1.1 (23). For nine more treatment episodes, the RECIST 1.1 response was not assessed through direct tumor measurement but abstracted from the treating physician notes, due to unavailability of outside scans to review. In accordance with RECIST 1.1, the disappearance of all lesions was considered as a complete response (CR), a reduction in the maximum tumor diameters after treatment ≥30% was considered a partial response (PR), an increase in the maximum tumor diameters ≥20% or the appearance of new lesions was considered disease progression (PD), while a change in maximum tumor diameters between −30% and +20% was considered stable disease (SD). Objective response rate (ORR) was defined as the proportion of patients achieving CR and PR out of all the patients treated, while the disease control rate (DCR) was defined as the proportion of patients that achieved PR, CR, or SD out of all the treated patients.

Outcomes measured included overall survival (OS), clinical progression-free survival from first-line treatment (first-line cPFS), and clinical progression-free survival combined for all treatment episodes regardless of line of therapy (combined cPFS). OS was measured from the time of pathologically confirmed diagnosis to death from any cause or was censored at the time of last follow-up. cPFS was measured from the start date of a new treatment until the date of the next clinically documented progression, as reported in the treating physician's notes. Disease progression was defined as either worsening radiographic tumor burden, not necessarily based on RECIST 1.1, or as worsening symptoms requiring the start of another line of therapy. When a treatment was stopped or changed because of patient's or physician's preference, because of toxicity, or for unknown reasons, patients were censored at the last follow-up or at the time of the start of the next treatment line.

As far as treatment doses, everolimus was given orally at a dose of 5 mg daily, temsirolimus was given intravenously at a dose of 25 mg once a week, sirolimus was given orally at a dose between 1 and 5 mg daily adjusted to target a therapeutic drug plasma level of 5–15 ng/mL, nab-sirolimus was given intravenously at a dose of 100 mg/m^2^ on days 1 and 8 of a 21-day cycle, olaparib was given orally at a dose of 300 mg twice daily, and pazopanib was given orally at a dose of 400 mg daily. Cytotoxic chemotherapy and immune checkpoint inhibitors (ICI) were administered according to standard dosing regimens.

### Statistical Analysis

Patient characteristics were summarized through frequency tables. Categorical variables were reported as proportions, while continuous variables were reported as median and range. Differences between categorical variables, including ORR and DCR, were measured through the Fisher exact test.

For survival analysis, the Kaplan–Meier method was used to create the survival curves and calculate the survival proportions as well as the median survival time with associated 95% confidence intervals (CI) where applicable. The log-rank test was used to calculate differences between the survival curves. We performed both a univariable and a multivariable Cox proportional hazards (PH) analysis for both OS and cPFS to calculate the HRs with associated 95% CIs. For OS, the variables included in the univariable Cox PH analysis were first-line treatment, sex, primary tumor site (uterine or extrauterine), age at diagnosis, *TSC1*/*TSC2* and *TP53* mutational status, TFE3 positivity (detected through either FISH or IHC), number of lines of therapy, histology (malignant PEComa vs. other), and history of tuberous sclerosis complex. The same covariates were included in the Cox PH model of first-line and combined cPFS, with the addition of the adjuvant treatment variable, to control for the longer PFS associated with treatments administered in the adjuvant setting. Also, for combined cPFS, given that multiple treatment episodes associated with the same patients are not independent events, a frailty covariate was included in the Cox PH model to control for random patient effect. For OS, only the variables that showed a significant *P* value in the univariable Cox PH analysis were included in the multivariable analysis together with the treatment variable. The same was true for first-line and combined cPFS, with the addition of the adjuvant treatment variable and the frailty covariate (only included for combined cPFS).

**FIGURE 1:**
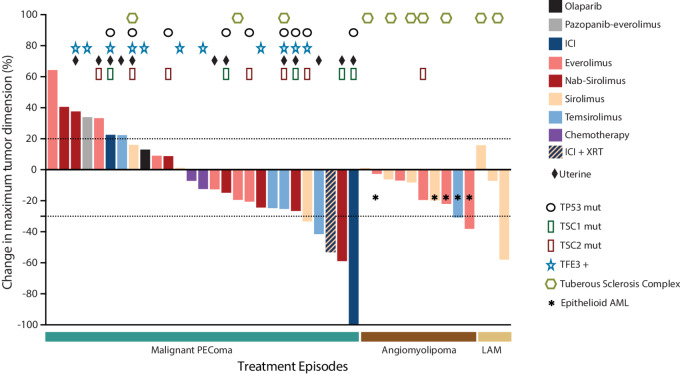
Best change in maximum tumor dimension for treatment episodes regardless of line of therapy. Waterfall plot shows the percent change in the sum of maximum tumor diameters pretreatment and posttreatment for the 40 treatment episodes for which radiographic imaging was directly available for assessment of response based on RECIST 1.1. The bars are color coded on the basis of the type of treatment. Information about TSC1/TSC2 and TP53 mutational status is shown for each patient, where available, and as described in the legend. Patients with epithelioid AML are indicated with an asterisk while those with a diagnosis of tuberous sclerosis complex are indicated with a hexagon. TFE3 + indicates both patients with TFE3 gene rearrangement or amplification on FISH as well as those with TFE3 protein overexpression on IHC. AML: angiomyolipoma; ICI: immune checkpoint inhibitors; LAM: lymphangioleiomyomatosis; PEComa: perivascular epithelioid cell tumors; XRT: radiotherapy.

For the 40 treatment episodes with available imaging and direct measurement of tumor dimensions, a waterfall plot showing the maximum change in tumor dimension pretreatment and posttreatment was created. All statistical analysis was performed in R version 4.2.2. The waterfall plot in Fig. 1 and the bar graph in Supplementary Fig. S2 were created with GraphPad Prism version 9.3.1 (GraphPad Software). *P* values ≤0.05 were considered significant. Correction for multiple hypothesis testing was not undertaken given the exploratory and hypothesis-generating nature of this study.

### Ethics Statement

The study was conducted according to the guidelines of the Declaration of Helsinki and was approved by Stanford University Institutional Review Board (IRB34465, approved on May 31, 2022). Patient consent was waived because of retrospective nature of the study.

### Data Availability Statement

The data generated in this study are available upon request from the corresponding author.

## Results

### Patient and Treatment Characteristics

A total of 29 patients were included in the study and their baseline characteristics are summarized in Table 1. Most patients were female (21/29, 72.4%), and the median age at diagnosis was 39 years old (range, 5–71). The most common histotype was malignant PEComa (17/29, 58.6%), followed by epithelioid AML (5/29, 17.2%), AML (4/29, 13.7%), and LAM (3/29, 10.3%). In those with malignant PEComa, the most common primary site was the uterus (9/17, 52.9%), while in patients with epithelioid AML/AML, the primary site was more often the kidney (8/10, 88.9%), and patients with LAM all had retroperitoneal involvement (3/3, 100%). Most patients had localized disease at diagnosis (22/29, 75.9%). For those with metastatic disease, the most common site of metastases was the lungs (8/29, 27.6%). For patients with tumor genetic testing available (Supplementary Fig. S2), the most common mutations were *TSC2* (5/11, 45.4%), and *TP53* (5/11, 45.4%), followed by *TSC1* (4/11, 36.4%). A total of 6 patients (20.7%) showed TFE3 positivity, that was defined as the presence of at least one between TFE3 protein overexpression detected on IHC, and *TFE3* gene amplification or rearrangement detected through FISH (24, 25). Of the patients defined as TFE3 positive, 2 were positive only on IHC, 1 was positive only on NGS with no FISH available, 1 was positive only on FISH with no NGS available, 1 was positive on both FISH and IHC with no NGS available, with only 1 patient being positive on both NGS, FISH and IHC (Supplementary Table S2). Of the patients defined as TFE3 negative, absence of TFE3 amplification could be confirmed by IHC only in 1 patient, by NGS and IHC in 1 patient, and by NGS only in another patient. For all the other patients, IHC staining, NGS or FISH was not available. Most patients had surgery of the primary tumor (22/29, 75.9%), with adjuvant or neoadjuvant systemic therapy, and most received only one line of systemic therapy (16/29, 55.2%). The most common systemic treatments were mTOR inhibitors (40/59 episodes, 67.8%), followed by cytotoxic chemotherapy (9/59, 15.4%), and ICIs (5/59, 8.4%; Supplementary Fig. S3). As far as mTOR inhibitors, these included everolimus (17/59, 28.8%), sirolimus (11/59, 18.6%), nab-sirolimus (7/59, 11.9%), and temsirolimus (5/59, 8.5%). In terms of cytotoxic chemotherapy regimens, these included anthracycline-based regimens (*n* = 3), carboplatin-paclitaxel (*n* = 2), and gemcitabine-docetaxel (*n* = 4). Median follow-up was 4.4 years (range, 0.4–18.0 years). For patients with localized disease at presentation, the median time to needing treatment from diagnosis was 12.5 months (range, 0–176.3 months).

**TABLE 1:** Baseline patient characteristics

Characteristics	Total (*N* = 29) *N* (%)	Malignant PEComa *N* (%)	Epithelioid AML/AML *N* (%)	LAM *N* (%)
Sex				
Female	21 (72.4)	13 (76.5)	6 (66.7)	2 (66.7)
Male	8 (27.6)	4 (23.5)	3 (33.3)	1 (33.3)
Age (years)				
Median, range	39 (5–71)	48 (5–71)	37 (22–69)	29 (23–39)
Histology				
Malignant PEComa	17 (58.6)			
Epithelioid AML	5 (17.2)			
AML	4 (13.7)			
LAM	2 (6.9)			
Primary site				
Kidney	10 (34.5)	2 (11.8)	8 (88.9)	0
Uterus	9 (31.0)	9 (52.9)	0	0
Retroperitoneum	5 (17.2)	1 (5.9)	1 (11.1)	3 (100.0)
Pelvis	1 (3.4)	1 (5.9)	0	0
Ovaries	1 (3.4)	1 (5.9)	0	0
Colon	1 (3.4)	1 (5.9)	0	0
Bladder	1 (3.4)	1 (5.9)	0	0
Trunk	1 (3.4)	1 (5.9)	0	0
Primary tumor resected				
Yes	22 (75.9)	17 (100)	5 (55.6)	0
No	7 (24.1)	0	4 (44.4)	3 (100)
Metastatic at diagnosis				
Yes	7 (24.1)	5 (29.4)	2 (22.2)	0
No	22 (75.9)	12 (70.6)	7 (77.8)	3 (100)
Locally advanced				
Yes	13 (44.8)	9 (52.9)	1 (11.1)	3 (100)
No	16 (55.2)	8 (47.1)	8 (88.9)	0
Metastases site				
Lung	8 (27.6)	7 (36.8)	1 (20.0)	—
Abdominal cavity	4 (13.8)	3 (15.8)	1 (20.0)	—
Spine	4 (13.8)	3 (15.8)	1 (20.0)	—
Liver	3 (10.3)	2 (10.5)	1 (20.0)	—
Pelvis	3 (10.3)	3 (15.8)	0	—
Lymph nodes	2 (6.9)	1 (5.3)	1 (20.0)	—
Tuberous sclerosis				
Yes	8 (27.6)	2 (11.8)	4 (44.4)	2 (66.7)
No	21 (72.4)	15 (88.2)	5 (55.6)	1 (33.3)
Ethnicity				
Caucasian	18 (62.1)	12 (70.6)	4 (44.4)	2 (66.7)
Hispanic/Latino	7 (24.1)	4 (23.5)	3 (33.3)	0
Asian	4 (13.8)	1 (5.9)	2 (22.2)	1 (33.3)
ECOG				
0	20 (69.0)	11 (64.7)	7 (77.8)	3 (100)
1	9 (31.0)	6 (35.3)	2 (22.2)	0
Primary tumor size (cm)				
Median (range)	12 (4.2–31.0)	10 (7.5–24.0)	12.1 (5.5–31.0)	13 (4.2–13.9)
Missing	4	4	0	0
Lines of therapy				
1	16 (55.2)	6 (35.3)	7 (77.8)	3 (100)
2	6 (20.7)	5 (29.4)	1 (11.1)	0
≥3	7 (24.1)	6 (35.3)	1 (11.1)	0
Follow-up time (years)				
Median (range)	4.4 (0.4–18.0)	4.4 (1.3–17.6)	4.4 (0.4–18.0)	7.9 (2.8–11.1)
Mutational burden (mut/Mb)				
Median, range	2.5 (1.0–17.0)	2.5 (1.0–17.0)	—	—
Missing	21	9	9	3
Status at last follow-up				
Alive	19 (65.5)	9 (52.9)	7 (77.8)	3 (100)
Deceased	10 (34.5)	8 (47.1)	2 (22.2)	0

Abbreviations: AML, angiomyolipoma; ECOG, Eastern cooperative oncology group; LAM, lymphangioleiomyomatosis; mut/Mb, mutations per megabase; PEComa, perivascular epithelioid cell tumors.

### Response Assessment

The best change in maximum tumor dimension for the 40 treatment episodes with available imaging data is reported in a waterfall plot in Fig. 1. The only CR was observed in a patient with malignant PEComa that was treated with pembrolizumab. Of note, this patient had a tumor mutational burden (TMB) of 17 mutations per megabase (mut/Mb). Regarding the other 2 patients with malignant PEComa treated with ICIs, both showed a TMB ≤2 mut/Mb, and of these only the patient that received pembrolizumab with concomitant radiotherapy obtained a PR, while the other patient, treated with ipilimumab-nivolumab, showed PD. The ORR and DCR for the 49 treatment episodes with available RECIST 1.1 response assessment, regardless of treatment line, are reported in Supplementary Table S3.

There was no difference in best DCR between patients treated with mTOR inhibitors and those that received other systemic therapies, both in the whole cohort (77.8% for *n* = 36 vs. 69.2% for *n* = 13, *P* = 0.70), in malignant PEComa only (63.3% for *n* = 22 vs. 66.7 for *n* = 22, *P* = 0.99), and in AML/LAM (*P* = 0.99; Supplementary Table S4). The same was true for ORR, with similar results between mTOR inhibitors and other treatments, in all the patients (16.7% vs. 15.4%, *P* = 0.99), in malignant PEComa only (13.6% vs. 16.7%, *P* = 0.99), and in AML/LAM only (*P* = 0.99). We also compared patients with malignant PEComa treated with nab-sirolimus with those that received other mTOR inhibitors and found no difference in either ORR or DCR.

We then compared ORR and DCR based on TFE3 positivity as well as on *TP53* and *TSC1*/*TSC2* mutational status (Supplementary Table S5). Regardless of type or line of therapy, we found no difference in DCR between TFE3 positive (*n* = 12) and negative (*n* = 37) patients (66.7% vs. 78.4%, *P* = 0.45), between those with (*n* = 9) and without (*n* = 40) *TP53* mutations (77.8% vs. 75.0%, *P* = 0.99), between those with (*n* = 17) and without (*n* = 32) *TSC1* or *TSC2* mutations (70.6% vs. 78.1%, *P* = 0.72), and between those with (*n* = 7) and without (*n* = 42) *TSC2* mutations only (71.4% vs. 76.2%, *P* = 0.99). Similar results were observed for ORR, with no difference between TFE3-positive and -negative patients (16.7% vs. 16.2%, *P* = 0.99), between patients with and without *TP53* mutations (22.2% vs. 15.0% *P* = 0.99), between those with and without *TSC1*/*TSC2* mutations (17.6% vs. 15.6%, *P* = 0.99), and between those with and without *TSC2* mutations only (14.3% vs. 16.7%, *P* = 0.99). Similar results were observed when considering only patients treated with mTOR inhibitors.

We also found that patients with uterine tumors had a lower DCR compared with those with an extrauterine location, both when considering all types of treatment (50.0% for *n* = 18 vs. 90.3% for *n* = 31, *P* = 0.004), and when considering mTOR inhibitors only (50.0% for *n* = 12 vs. 91.7% for *n* = 24, *P* = 0.009). When examining only patients with malignant PEComa, there was a similar trend toward worse DCR for those with a primary uterine site, both when considering all treatments and only mTOR inhibitors, but statistical significance was not reached (Supplementary Table S5). Of note, 33.3% of patients with uterine PEComas had a *TP53* mutation compared with 10.0% of those with extrauterine tumors (*P* = 0.28), while 33.3% showed TFE3 positivity compared with 15.0% of patients with extrauterine tumors (*P* = 0.33). Also, among patients with malignant PEComas, there was no difference between those with uterine and extrauterine tumors in terms of frequency of *TP53* mutations (33.3% vs. 25.0%, *P* = 0.99) or TFE3 positivity (33.3% vs. 37.5%, *P* = 0.99). Finally, coexisting *TP53* mutation and TFE3 positivity was found in 2 patients with uterine PEComas versus only 1 patient with extrauterine disease.

### OS

**FIGURE 2:**
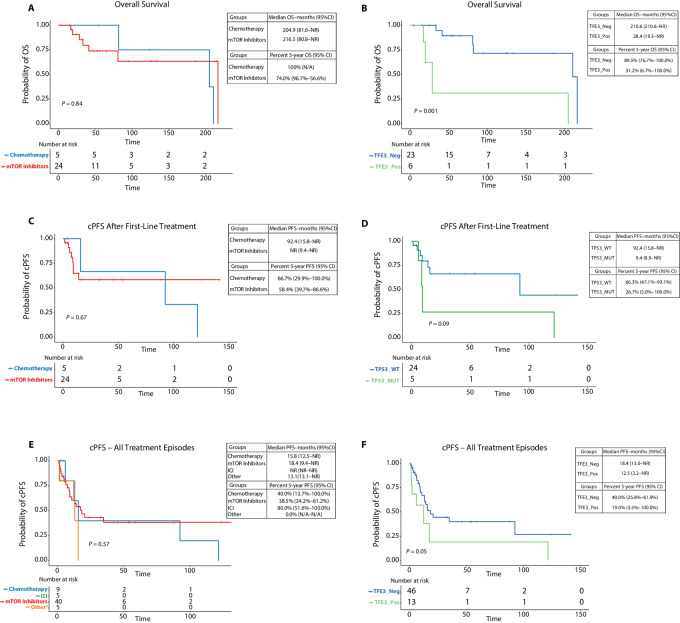
OS and cPFS. **A,** Kaplan–Meier curve shows OS in patients treated with first-line chemotherapy versus mTOR inhibitors. **B,** Kaplan–Meier curve shows OS of patients with TFE3 positivity as detected through either IHC or FISH compared with those that were TFE3 negative. **C,** Kaplan–Meier curve shows cPFS from first-line treatment in patients that received chemotherapy compared with those treated with mTOR inhibitors. **D,** Kaplan–Meier curve shows cPFS from first-line treatment in patients with (TP53_MUT) and without (TP53_WT) a TP53 mutation. **E,** Kaplan–Meier curve showing cPFS for all treatment episodes regardless of line of therapy, and comparing chemotherapy, ICI, mTOR inhibitors, and other treatments. **F,** Kaplan–Meier curve shows cPFS for all treatment episodes regardless of line of therapy in patients that are TFE3 positive versus those that are TFE3 negative. log-rank *P* values are shown. Other^a^ included: olaparib (*n* = 1), pazopanib (*n* = 1), pazopanib-everolimus (*n* = 1), anastrozole (*n* = 1), and levantinib-everolimus (*n* = 1). NR, not reachable.

Median OS was 204.9 months (95% CI: 81.6–not reached) for all the patients in the study, 81.6 months (95% CI: 80.8–not reached) for those with malignant PEComa, and 216.5 months (95% CI: not reached) for those with AML and LAM combined (Supplementary Table S6; Supplementary Fig. S4A). There was no difference in median OS between patients treated with first-line chemotherapy (204.9 months, 95% CI: 81.6–not reached) and those that received mTOR inhibitors as first-line including nab-sirolimus (*n* = 4), everolimus (*n* = 9), sirolimus (*n* = 8), and temsirolimus (*n* = 3; 216.5 months, 95% CI: 80.8–not reached, *P* = 0.84; Fig. 2A). TFE3 positivity correlated with increased risk of death (HR: 8.0, 95% CI: 1.9–34.5, *P* = 0.005; Table 2) and shorter median OS (28.4 vs. 210.6 months, *P* = 0.001; Fig. 2B). Metastatic disease at diagnosis also correlated with increased risk of death (HR: 6.7, 95% CI: 1.1–41.2, *P* = 0.03) and shorter median OS (41.1 vs. 210.6 months, *P* = 0.02). Presence of *TSC1*/*TSC2* mutations or of a *TP53* mutation did not correlate with shorter median OS or with increased risk of death (Supplementary Fig. S4B and S4C). Other than TFE3 positivity and primary metastatic disease, the other variables that correlated with worse OS in the univariable analysis were receiving two lines of therapy (HR: 14.4, 95% CI: 2.3–90.0, *P* = 0.004), malignant PEComa histology (HR: 8.1, 95% CI: 1.0–65.3, *P* = 0.05). In the multivariable analysis, TFE3 positivity still correlated with higher risk of death (HR: 17.4, 95% CI: 1.5–204.1, *P* = 0.02). Similar results were observed when analyzing only patients with malignant PEComa (Supplementary Table S7; Supplementary Fig. S5).

**TABLE 2:** Cox PH analysis for OS

		Univariable			Multivariable	
Variables	*N* Episodes (%)	HR (95%CI)	*P* (Cox-Wald)	*P* (log-rank)	HR (95%CI)	*P* (Cox-Wald)
Sex						
Male	8 (27.6)	0.4 (0.05–3.1)	0.4	0.3		
Female	21 (72.4)	—	—			
Primary site						
Uterine	9 (31.0)	2.5 (0.6–9.9)	0.2	0.3		
Extrauterine	20 (68.9)	—	—			
Age, years (median, range)	39 (5–71)	1.0 (0.9–1.1)	0.3	0.3		
Metastatic at diagnosis						
Yes	7 (24.1)	6.7 (1.1–41.2)	**0.03**	**0.02**	5.1 (0.6–40.9)	0.1
No	22 (75.9)					
TSC mutation						
*TSC2* mutated	5 (17.2)	0.8 (0.07–9.8)	0.9	0.57		
*TSC1*/*TSC2* wild type	20 (68.9)	0.4 (0.05–3.7)	0.4			
*TSC1* mutated	4 (13.8)	—	—			
*TP53*						
Mutated	5 (17.2)	3.8 (0.9–16.4)	0.07	0.06		
Wild type	24 (82.8)	—	—			
Lines of therapy						
1	17 (58.6)	—	—		—	—
2	5 (17.2)	14.4 (2.3–90.0)	**0.004**	**0.0004**	4.5 (0.4–49.3)	0.2
≥3	7 (24.1)	1.1 (0.1–6.5)	0.9		0.2 (0.01–3.8)	0.3
TFE3						
Negative	23 (79.3)	—	—		—	—
Positive	6 (20.7)	8.0 (1.9–34.5)	**0.005**	**0.001**	17.4 (1.5–204.1)	0.02
Histology						
Malignant PEComa	17 (58.6)	8.1 (1.0–65.3)	0.05	**0.02**	3.3 (0.1–82.0)	0.5
LAM/AML/Epithelioid AML	12 (41.4)	—	—		—	—
History of tuberous sclerosis						
Yes	8 (27.6)	0.3 (0.04–2.7)	0.3	0.3		
No	21 (72.4)	—	—			
Treatment (first line)						
mTOR inhibitors	24 (82.8)	0.9 (0.2–3.6)	0.8	0.8	1.1 (0.04–29.1)	0.9
Chemotherapy	5 (17.2)	—	—			

Abbreviations: AML, angiomyolipoma; LAM, lymphangioleiomyomatosis; mTOR, mammalian target of rapamycin; PEComa, perivascular epithelioid cell tumors.

### cPFS

We performed cPFS analysis on two levels: on a patient level, considering only first-line treatments for the 29 patients in the study (first-line cPFS), and on a treatment level, considering all treatment episodes regardless of line of therapy (combined cPFS).

Median first-line cPFS for all patients in the study was 92.4 months (95% CI: 14.1–not reached; Supplementary Table S8; Supplementary Fig. S6), with no difference between those treated with chemotherapy (92.4 months, 95% CI: 15.8–not reached) and those that received mTOR inhibitors (not reached, 95% CI: 9.4–not reached, *P* = 0.67; Fig. 2C), or between patients with localized disease (92.4 months, 95% CI: 15.8–not reached) and those with metastases at diagnosis (9.4 months, 95% CI: 6.0–not reached, *P* = 0.1). There was a nonsignificant trend for shorter median first-line cPFS in those with *TP53* mutations (9.4 months, 95% CI: 8.9–not reached) compared with those without (92.4 months, 95% CI: 15.8–not reached, *P* = 0.09; Fig. 2D). TFE3 positivity did not correlate with higher risk of progression after first-line therapy (HR: 2.0, 95% CI: 0.5–8.0, *P* = 0.3; Table 3), while there was a nonsignificant trend for lower risk of progression after first-line treatment in patients without *TSC1*/*TSC2* mutations (HR: 0.2, 95% CI: 0.04–1.2, *P* = 0.07). The only variables associated with higher progression risk after first-line treatment in the univariable analysis were age at diagnosis (HR: 1.1, 95% CI: 1.0–1.2, *P* = 0.001), receiving two (HR: 18.4, 95% CI: 3.1–109.7, *P* = 0.001) or more than three lines of therapy (HR: 4.6, 95% CI: 1.0–20.7, *P* = 0.04), and malignant PEComa histology (HR: 9.6, 95% CI: 1.2–76.8, *P* = 0.03). Age at diagnosis was the only variable associated with increased risk of progression after first-line therapy in the multivariable analysis (HR: 1.1, 95% CI: 1.0–1.1, *P* = 0.006). Again, similar results for first-line cPFS were observed in patients with malignant PEComa when analyzed separately (Supplementary Tables S9 and S10).

**TABLE 3:** Cox PH analysis for PFS from first-line treatment

		Univariable			Multivariable	
Variables	*N* Episodes (%)	HR (95% CI)	*P* (Cox-Wald)	*P* (log-rank)	HR (95% CI)	*P* (Cox-Wald)
Sex						
Male	8 (27.6)	0.5 (0.1–2.5)	0.4	0.4		
Female	21 (72.4)	—	—			
Primary site						
Uterine	9 (31.0)	2.5 (0.7–8.5)	0.1	0.1		
Extrauterine	20 (68.9)	—	—			
Age, years (median, range)	39 (5–71)	1.1 (1.0–1.2)	**0.001**	**0.0001**	1.1 (1.0–1.1)	**0.006**
Metastatic at diagnosis						
Yes	7 (24.1)	2.7 (0.7–10.3)	0.1	0.1		
No	22 (75.9)					
TSC mutation						
*TSC2* mutated	5 (17.2)	0.5 (0.08–3.6)	0.5	0.1		
*TSC1*/*TSC2* Wild type	20 (68.9)	0.2 (0.04–1.2)	0.07			
*TSC1* mutated	4 (13.8)	—	—			
*TP53*						
Mutated	5 (17.2)	2.8 (0.8–10.1)	0.1	0.09		
Wild type	24 (82.8)	—	—			
Lines of therapy						
1	17 (58.6)	—	—		—	—
2	5 (17.2)	18.4 (3.1–109.7)	**0.001**	**0.0004**	3.7 (0.4–31.3)	0.2
≥3	7 (24.1)	4.6 (1.0–20.7)	**0.04**		5.2 (0.3–89.8)	0.2
TFE3						
Negative	23 (79.3)	—	—			
Positive	6 (20.7)	2.0 (0.5–8.0)	0.3	0.3		
Histology						
Malignant PEComa	17 (58.6)	9.6 (1.2–76.8)	**0.03**	**0.01**	4.9 (0.4–61.2)	0.2
LAM/AML/Epithelioid AML	12 (41.4)	—	—		—	—
History of tuberous sclerosis						
Yes	8 (27.6)	0.3 (0.04–2.7)	0.3	0.3		
No	21 (72.4)	—	—			
Treatment (first line)						
mTOR inhibitors	24 (82.8)	0.7 (0.2–2.9)	0.7	0.7	0.5 (0.03–8.9)	0.6
Chemotherapy	5 (17.2)	—	—		—	—
Adjuvant treatment						
Yes	5 (17.2)	1.6 (0.4–6.1)	0.5	0.5	0.2 (0.01–4.2)	0.3
No	24 (82.8)	—				

Abbreviations: AML, angiomyolipoma; LAM, lymphangioleiomyomatosis; mTOR, mammalian target of rapamycin; PEComa, perivascular epithelioid cell tumors.

Median combined cPFS in the whole cohort was 15.8 months (95% CI: 12.5–not reached; Supplementary Table S11; Supplementary Fig. S7), with no difference between patients treated with chemotherapy, mTOR inhibitors, ICIs, and other treatments in the log-rank analysis (Fig. 2E). Also, there was no difference in median combined cPFS between patients with localized disease (17.4 months, 95% CI: 13.0–not reached) and those with metastases at diagnosis (9.4 months, 95% CI: 6.0–not reached *P* = 0.2). There was a nonsignificant trend for shorter median combined cPFS in patients that were TFE3 positive (12.5 months, 95% CI: 2.2–not reached) compared with those that were TFE3 negative (18.4 months, 95% CI: 13.0–not reached, *P* = 0.05; Fig. 2F). Similarly, patients with *TP53* mutations tended to have shorter median combined cPFS compared with those that were *TP53* wild type (*P* = 0.09), and those with primary uterine tumors tended to have shorter median combined cPFS than those with extrauterine tumors (*P* = 0.09), even though statistical significance was not met in both cases (Supplementary Fig. S7). Variables that correlated with shorter combined cPFS in the univariable analysis were age at diagnosis (HR: 1.1, 95% CI: 1.0–1.1, *P* = 0.004), receiving two (HR: 13.8, 95% CI: 3.4–56.4, *P* = 0.0003) or more than three lines of therapy (HR: 7.9, 95% CI:2.3–27.3, *P* = 0.0009), and malignant PEComa histology (HR: 5.6, 95% CI: 1.7–18.7, *P* = 0.005; Table 4). Of these, malignant PEComa histology (*P* = 0.02), receiving three or more lines of therapy (*P* = 0.04), and age at diagnosis (*P* = 0.02) retained their association with worse combined cPFS in the multivariable analysis. Also, in the multivariable analysis, mTOR inhibitors showed a nonsignificant trend toward reduced progression risk (HR: 0.2, 95% CI: 0.04–1.2, *P* = 0.07). We also measured cPFS for each of the mTOR inhibitors separately, including everolimus (*n* = 17), sirolimus (*n* = 11), nab-sirolimus (*n* = 7), and temsirolimus (*n* = 5), and compared these with chemotherapy, ICI, and other treatments (Supplementary Fig. S8). While there was no difference in combined cPFS among the different treatments in the log-rank analysis (*P* = 0.19), both nab-sirolimus (HR: 0.1, 95% CI: 0.01–0.9, *P* = 0.04), and sirolimus (HR: 0.04, 95% CI: 0.003–0.6, *P* = 0.01) correlated with reduced progression risk in the multivariable Cox analysis.

**TABLE 4:** Cox PH analysis for PFS for all treatment episodes combined, regardless of line of therapy

		Univariable			Multivariable	
Variables	*N* Episodes (%)	HR (95% CI)	*P* (Cox-Wald)	*P* (log-rank)	HR (95%CI)	*P* (Cox-Wald)
Sex						
Male	11 (18.6)	0.5 (0.1–1.6)	0.2	0.3		
Female	48 (81.4)	—	—			
Primary site						
Uterine	25 (42.4)	1.9 (0.9–3.9)	0.1	0.09		
Extrauterine	34 (57.6)	—	—			
Age, years (median, range)	39 (5–71)	1.1 (1.0–1.1)	**0.004**	**0.003**	1.1 (1.0–1.1)	**0.02**
Metastatic at diagnosis						
Yes	13 (22.0)	1.9 (0.7–5.4)	0.2	0.2		
No	46 (78.0)					
TSC mutation						
*TSC2* mutated	10 (16.9)	0.7 (0.2–2.9)	0.6	0.4		
*TSC1*/*TSC2* wild type	38 (64.5)	0.5 (0.1–1.5)	0.2			
*TSC1* mutated	11 (18.6)	—	—			
*TP53*						
Mutated	10 (16.9)	2.3 (0.8–6.1)	0.1	0.09		
Wild type	49 (83.1)	—	—			
Lines of therapy						
1	18 (30.6)	—	—		—	—
2	10 (16.9)	13.8 (3.4–56.4)	**0.0003**	**0.00007**	2.9 (0.3–25.0)	0.3
≥3	31 (52.5)	7.9 (2.3–27.3)	**0.0009**		7.0 (1.1–44.2)	**0.04**
TFE3						
Negative	46 (77.9)	—	—			
Positive	13 (22.1)	2.7 (0.9–7.7)	0.06	0.05		
Frailty (Patient ID)	—	—	—		—	—
Histology						
Malignant PEComa	42 (71.2)	5.6 (1.7–18.7)	**0.005**	**0.002**	11.4 (1.4–96.1)	**0.02**
LAM/AML/Epithelioid AML	17 (28.8)	—	—		—	—
History of tuberous sclerosis						
Yes	11 (18.6)	0.3 (0.04–2.7)	0.3	0.3		
No	48 (81.4)	—	—			
Treatment (all lines)						
mTOR inhibitors	40 (67.8)	0.8 (0.3– 2.3)	0.7	0.6	0.2 (0.04–1.2)	0.07
Other*^a^*	5 (8.4)	1.9 (0.4–8.6)	0.4		0.3 (0.04–2.9)	0.3
ICI	5 (8.4)	1.5 (0.2–13.7)	0.7		0.2 (0.02–3.3)	0.3
Chemotherapy	9 (15.4)	—	—		—	—
Adjuvant treatment						
Yes	7 (11.9)	0.3 (0.07–1.0)	0.051	0.6	0.04 (0.007–0.3)	**0.0007**
No	52 (88.1)	—				

Abbreviations: AML, angiomyolipoma; LAM, lymphangioleiomyomatosis; mTOR, mammalian target of rapamycin; ICI: Immune check-point inhibitors; PEComa, perivascular epithelioid cell tumors.

*^a^* Other: olaparib (*n* = 1), pazopanib (*n* = 1), pazopanib-everolimus (*n* = 1), anastrozole (*n* = 1), and levantinib-everolimus (*n* = 1).

Also, when analyzing only patients with malignant PEComa, we found no significant associations for combined cPFS (Supplementary Table S12; Supplementary Fig. S9), as well as no difference in combined cPFS between patients treated with mTOR inhibitors considered separately, ICI, chemotherapy, and other treatments (*P* = 0.93, log-rank; Supplementary Fig. S10).

## Discussion

In this study, we report on the outcomes of what, to our knowledge, is the largest single-institution cohort of patients with advanced PEComas receiving systemic therapy, including both patients with malignant PEComa, AML, epithelioid AML, and LAM.

The median OS from first-line therapy for all the patients in the study was 18 years (median follow-up of 4.4 years), and as expected it tended to be shorter in patients with malignant PEComa (6.8 years) than in those with AML, epithelioid AML, and LAM combined (18 years). Overall, our patients showed longer OS than what observed in a prior retrospective study that included both patients with malignant PEComa and epithelioid AML (median OS of 2.5 years with median follow-up of 2.7 years), and in a case series of 10 patients with malignant PEComa (median OS of 2.4 years with median follow-up of 1.9 years; refs. 17, 10).

When comparing different systemic therapies, we found no difference in OS between patients treated with first-line chemotherapy versus those that received mTOR inhibitors, and the same was true for first-line cPFS, both in the cohort as a whole and in patients with malignant PEComa only.

As far as combined cPFS, mTOR inhibitors showed a nonsignificant trend for better cPFS compared with chemotherapy, ICIs, and other treatments including pazopanib and levantinib—alone or in combination with everolimus—and the PARP inhibitor olaparib. Among the mTOR inhibitors, nab-sirolimus and sirolimus correlated with better combined cPFS compared with all other treatments. However, these results were not replicated when analyzing patients with malignant PEComa separately. Our findings contrast with what shown by Sanfilippo and colleagues, where chemotherapy resulted in shorter PFS than mTOR inhibitors, including sirolimus, everolimus, and temsirolimus (17). It must be noted though that combined cPFS was longer in our study for both chemotherapy (15.8 vs. 3.4 months for gemcitabine-based regimens and 3.2 months for anthracycline based regimens) and mTOR inhibitors (18.4 vs. 9 months) when compared with the study from Sanfilippo and colleagues. These results are most likely explained by heterogeneity in the patient populations, because our study included not only patients with malignant PEComa and epithelioid AML, but also patients with AML and LAM which do have a more indolent course. Patients with AML and LAM included in our study received systemic therapy for symptomatic unresectable disease.

Considering each mTOR inhibitor separately, the median PFS in the whole cohort was respectively 14.1 months for everolimus, 9.4 months for nab-sirolimus, and 7.6 months for temsirolimus, while it was not reached for sirolimus. This is similar to what observed by Sanfilippo and colleagues when considering all mTOR inhibitors together (median PFS: 9 months; ref. 17). Also, as far as nab-sirolimus, the median cPFS of 9.4 months observed in our study is comparable with the results of the AMPECT trial where median PFS was 10.6 months (16). Furthermore, it is worth mentioning that the ORR for patients treated with nab-sirolimus in our study was 14.3% versus 39% in the AMPECT trial. This discrepancy could be attributed to differences in patient populations between the studies, as well as variability in the measurement of radiological response due to the absence of centralized radiological review in our study. However, it is crucial to acknowledge the limitations of comparing a retrospective study, such as ours, to a prospectively defined study like the AMPECT trial, as well as the potential pitfalls of interstudy comparisons.

As far as tumor response, we found no difference in both DCR and ORR between mTOR inhibitors and other treatments combined. This was true both when considering the cohort as a whole and when analyzing malignant PEComas and other histotypes separately. Even though a direct comparison of tumor response between each treatment was not feasible given the size of our cohort, the ORR and DCR observed for each mTOR inhibitor, and the other treatment modalities considered separately appeared to be similar.

To our knowledge, this is one of the few reports on ICIs use in patients with malignant PEComa (26, 27). Interestingly, we observed a CR in a patient treated with pembrolizumab that also showed a TMB of 17 mut/Mb. This is consistent with what observed in other cancers where a high TMB, usually above 10 mut/Mb, correlates with a more favorable response to ICIs, likely due to an increase in neoantigens and consequently in tumor immunogenicity (28, 29). Of the two other patients in our cohort that were treated with ICIs and that had imaging available for direct assessment of response, one received ipilimumab-nivolumab with disease progression, and the other received pembrolizumab plus radiotherapy achieving a PR. Interestingly, both patients had TMB ≤2 mut/Mb, suggesting that, in patients with low mutational burden, radiotherapy might have a synergistic effect with ICIs as reported in previous preclinical and clinical studies (30, 31).

When considering the whole cohort, we found that patients with TFE3 positivity had worse OS and increased risk of death both in the univariable and multivariable Cox analyses, as well as a nonsignificant trend toward worse combined cPFS (*P* = 0.05). When analyzing patients with malignant PEComa only, we found a similar association between TFE3 and shorter OS, even though in this case TFE3 positivity was not significantly associated with worse OS in the Cox analysis, likely due to the small sample size (*n* = 17). We defined TFE3 positivity broadly as either *TFE3* gene rearrangement or amplification on FISH, or as TFE3 protein overexpression on IHC, because the consequence of these molecular changes is hypothesized to be an increase in TFE3 activity. Previous reports have shown that PEComas associated with *TFE3* gene rearrangements form a distinct molecular subtype which tends to have a more aggressive behavior (24, 25, 32). Also, *TFE3* rearrangements have been found to be mutually exclusive with *TSC2* mutations in PEComas which has been hypothesized to be responsible for the worse prognosis of these patients (25, 33). However, a recent report of an aggressive PEComa harboring a *TSC1* mutation coexisting with a *TFE3* gene amplification has challenged the dichotomy of this molecular classification (34). According to this finding, in our study, TFE3 overexpression on IHC coexisted with *TSC2* mutation in 2 patients, and *TFE3* gene amplification was found in a patient with a *TSC1* mutation. Overall, our results suggest a negative prognostic role in PEComas for TFE3 positivity broadly defined as either gene amplification or rearrangement, or only as protein overexpression. Together with prior reports, our results indicate that an increase in the activity of TFE3, not exclusively through *TFE3* gene translocation, might be responsible for the worse prognosis of this subtype of PEComas. Our results also suggest that mechanisms different from gene fusion might be responsible for the increased activity of TFE3 in PEComas. However, a major limitation of our study is that confirmation of TFE3 negativity by either IHC or NGS was available only for 3 patients, with molecular data regarding TFE3 overexpression not available for review in most patients. For this reason, further studies will be needed to confirm the prognostic role of TFE3 overexpression in PEComas, and to define the molecular basis for TFE3 overactivity in PEComas and how this could be exploited therapeutically.

In addition, in contrast to what shown in the AMPECT trial, in our study *TSC2* mutations did not correlate with better response, either when considering all treatments or mTOR inhibitors only, and patients with *TSC1*/*TSC2* mutations showed similar ORR and DCR to those without *TSC1*/*TSC2* mutations (16). It is possible that *TSC2* mutations might confer a favorable prognosis specifically to patients treated with nab-sirolimus rather than with other mTOR inhibitors. However, our study was not powered to answer this question and further larger studies including different types of mTOR inhibitors will be needed to address this issue.

Finally, we found that extrauterine PEComas showed higher DCR than uterine PEComas, similar to prior reports (17). This could be in part explained by the fact that most malignant PEComas in our study arose in the uterus, while none of the AML or LAM cases had a uterine location. However, even when considering only malignant PEComas, there was still a tendency toward higher DCR for extrauterine tumors. It is possible that, regardless of the histotype, there might be biological differences between uterine and extrauterine PEComas that could account for the observed differences in response.

## Conclusion

In summary, this study did not show a clear difference between mTOR inhibitors and chemotherapy, both in terms of OS, PFS, and tumor response. Together with evidence from prior studies, our results suggest that mTOR inhibitors should be the agents of choice in patients with PEComas, considering the better safety profile of these agents compared with cytotoxic chemotherapy. Also, we did not find a definitive superiority of either one of the mTOR inhibitors above the others, including the new agent nab-sirolimus, suggesting that other factors like availability and cost might be important considerations when choosing a specific agent from this class. Also, our results provide preliminary evidence that increased activity of TFE3, achieved through either gene amplification or rearrangement or through protein overexpression, might define a distinct class of PEComas characterized by worse prognosis and a more aggressive behavior. Finally, this study presents important limitations, including a retrospective design, the inclusion of patients treated at a single institution, the lack of molecular testing for most patients, and a small sample size. It should be emphasized that the small sample size can lead to overestimation of subtle differences observed in our cohort and have major implications for statistical significance. Larger prospective studies comparing specific agents, as well as larger retrospective multi-institutional reviews, will be needed to confirm our findings.

## Supplementary Material

Figure S1 Supplementary Figure S1 showing study flow diagram with details regarding the pathology cases review and patient selection process.

Figure S2 Figure S2 shows an Oncoplot of the mutations discovered in patients for which next-generation sequencing testing was available.

Figure S3 Figure S3 shows a bar graph with the number of treatment episodes in each disease group classified based on type and line of treatment.

Figure S4 Figure S4 shows Kaplan-Meier curves for Overall Survival in patients with PEComas and effect of TSC1/TSC2 and TP53 mutation on Overall Survival.

Figure S5 Figure S5 shows Kaplan-Meier Curves of Overall Survival for patients with Malignant PEComas treated with chemotherapy or mTOR inhibitors, and for patients with and without TP53 mutations, with and without TSC1/TSC2 mutations, and for those that are TFE3 positive versus TFE3 negative.

Figure S6 Figure S6 shows Kaplan-Meier curves for clinical PFS from first-line therapy in patients with PEComas, and specifically effect of TFE3 positivity, TSC1/TSC2 mutational status, and uterine versus extra-uterine location on clinical PFS.

Figure S7 Figure S7 shows Kaplan-Meier curves for combined PFS in patients with PEComas, specifically showing effect of TP53 and TSC1/TSC2 mutational status, as well as uterine vs extra-uterine location on combined PFS.

Figure S8 Figure S8 shows combined clinical progression-free survival (cPFS) in the whole cohort comparing each mTOR inhibitors to other treatments.

Figure S9 Figure S9 shows Kaplan-Meier curves for combined clinical PFS in patients with malignant PEComas. Specifically, figure shows effects on combined clinical PFS of TFE3 positivity, treatment type, and TP53 and TSC1/TSC2 mutational status.

Figure S10 Figure S10 shows a Kaplan-Meier curve that represents effects of different treatments on combined clinical PFS only in patients with malignant PEComa.

Table S1 Table S1 shows a a description of study design, main findings and conclusions for the most relevant references in the study regarding management of patients with PEComas.

Table S2 Table S2 shows the results of molecular testing available for each patient in the study.

Table S3 Table S3 shows the best overall response rate (ORR) and disease control rate (DCR) for different treatment types based on histology, tumor location, TFE3 positivity, as well as TSC1, TSC2, and TP53 mutational status.

Table S4 Table S4 shows the best disease control rate (DCR) and overall response rate (ORR) for mTOR inhibitors compared to other treatments based on histotype.

Table S5 Table S5 shows DCR and ORR based on biomarker status and primary tumor site, considering all treatment episodes, and mTOR inhibitors only.

Table S6 Table S6 shows median Overall Survival in months, as well as 5-year Overall Survival rate for the whole cohort.

Table S7 Table S7 shows results of Cox proportional hazard analysis for overall survival in patients with malignant PEComa only.

Table S8 Table S8 shows median clinical PFS from first-line therapy in months, as well as 5-year clinical PFS rate from first-line therapy for the whole cohort.

Table S9 Table S9 shows Cox proportional hazard analysis results for clinical progression-free survival from first-line therapy in patients with malignant PEComa.

Table S10 Table S10 shows median clinical PFS from first-line therapy in months, as well as 5-year clinical PFS rate from first-line therapy in patients with malignant PEComa.

Table S11 Table S11 shows median clinical PFS for all treatment episodes in months, as well as 5-year clinical PFS rate for all treatment episodes in the whole cohort.

Table S12 Table S12 shows results of Cox proportional hazard analysis for progression-free survival for all treatment episodes combined, regardless of line of therapy in patients with malignant PEComa only.

## References

[1] Folpe AL , Kwiatkowski DJ. Perivascular epithelioid cell neoplasms: pathology and pathogenesis. Hum Pathol 2010;41:1–15. 19604538 10.1016/j.humpath.2009.05.011

[2] Doyle LA , Argani P, Hornick JL. PEComa. In: WHO classification of tumors editorial board. Soft tissue and bone tumours. Lyon, France: International Agency for Research on Cancer; 2020. pp.312–4.

[3] Bonetti F , Pea M, Martignoni G, Zamboni G. PEC and sugar. Am J Surg Pathol 1992;16:307–8. 1599021 10.1097/00000478-199203000-00013

[4] Rule AD , Sasiwimonphan K, Lieske JC, Keddis MT, Torres VE, Vrtiska TJ. Characteristics of renal cystic and solid lesions based on contrast-enhanced computed tomography of potential kidney donors. Am J Kidney Dis 2012;59:611–8. 22398108 10.1053/j.ajkd.2011.12.022 PMC3328591

[5] Stacchiotti S , Frezza AM, Blay JY, Baldini EH, Bonvalot S, Bovée JVMG, . Ultra-rare sarcomas: a consensus paper from the Connective Tissue Oncology Society community of experts on the incidence threshold and the list of entities. Cancer 2021;127:2934–42. 33910263 10.1002/cncr.33618 PMC8319065

[6] Kenerson H , Folpe AL, Takayama TK. Activation of the mTOR pathway in sporadic angiomyolipomas and other perivascular epithelioid cell neoplasms. Hum Pathol 2007;38:1361–71. 17521703 10.1016/j.humpath.2007.01.028 PMC2722219

[7] Henske EP , Jóźwiak S, Kingswood JC, Sampson JR, Thiele EA. Tuberous sclerosis complex. Nat Rev Dis Primers 2016;2:16035. 27226234 10.1038/nrdp.2016.35

[8] Liu GY , Sabatini DM. mTOR at the nexus of nutrition, growth, ageing and disease. Nat Rev Mol Cell Biol 2020;21:183–203. 31937935 10.1038/s41580-019-0199-y PMC7102936

[9] Italiano A , Delcambre C, Hostein I, Cazeau AL, Marty M, Avril A, . Treatment with the mTOR inhibitor temsirolimus in patients with malignant PEComa. Ann Oncol 2010;21:1135–7. 20215136 10.1093/annonc/mdq044

[10] Benson C , Vitfell-Rasmussen J, Maruzzo M, Fisher C, Tunariu N, Mitchell S, . A retrospective study of patients with malignant PEComa receiving treatment with sirolimus or temsirolimus: the Royal Marsden Hospital experience. Anticancer Res 2014;34:3663–8. 24982384

[11] Wagner AJ , Malinowska-Kolodziej I, Morgan JA, Qin W, Fletcher CDM, Vena N, . Clinical activity of mTOR inhibition with sirolimus in malignant perivascular epithelioid cell tumors: targeting the pathogenic activation of mTORC1 in tumors. J Clin Oncol 2010;28:835–40. 20048174 10.1200/JCO.2009.25.2981 PMC4810029

[12] Dickson MA , Schwartz GK, Antonescu CR. Extrarenal perivascular epithelioid cell tumors (PEComas) respond to mTOR inhibition: clinical and molecular correlates. Int J Cancer 2013;132:1711–7. 22927055 10.1002/ijc.27800 PMC3558545

[13] Weeber F , Koudijs MJ, Hoogstraat M, Besselink NJM, Lieshout SV, Nijman IJ, . Effective therapeutic intervention and comprehensive genetic analysis of mTOR signaling in PEComa: a case report. Anticancer Res 2015;35:3399–403. 26026101

[14] Bissler JJ , McCormack FX, Young LR, Elwing JM, Chuck G, Leonard JM, . Sirolimus for angiomyolipoma in tuberous sclerosis complex or lymphan- gioleiomyomatosis. N Engl J Med 2008;358:140–51. 18184959 10.1056/NEJMoa063564 PMC3398441

[15] Bissler JJ , Kingswodd JC, Radzikowska E, Zonnenberg BA, Frost M, Belousova E, . Everolimus for angiomyolipoma associated with tuberous sclerosis complex or sporadic lymphangioleiomyomatosis (EXIST-2): a multicentrem randomised, double-blind, placebo-controlled trial. Lancet 2013;381:817–24. 23312829 10.1016/S0140-6736(12)61767-X

[16] Wagner AJ , Ravi V, Riedel RF, Ganjoo K, Van Tine BA, Chugh R, . nab-Sirolimus for patients with malignant perivascular epithelioid cell tumors. J Clin Oncol 2021;39:3660–70. 34637337 10.1200/JCO.21.01728 PMC8601264

[17] Sanfilippo R , Jones RL, Blay JY, Le Cesne A, Provenzano S, Antoniou G, . Role of chemotherapy, VEGFR inhibitors, and mTOR inhibitors in advanced perivascular epithelioid cell tumors (PEComas). Clin Cancer Res 2019;25:5295–300. 31217199 10.1158/1078-0432.CCR-19-0288

[18] Lee DW , Chang H, Kim YJ, Kim KM, Lee HJ, Lee JS. Sorafenib-induced tumor response in a patient with metastatic epithelioid angiomyolipoma. J Clin Oncol 2014;32:e42–5. 24449236 10.1200/JCO.2012.48.1960

[19] Machado I , Cruz J, Lavernia J, Rayon JM, Poveda A, Llombart-Bosch A. Malignant PEComa with metastatic disease at diagnosis and resistance to several chemotherapy regimens and targeted therapy (m-TOR inhibitor). Int J Surg Pathol 2017;25:543–9. 28459168 10.1177/1066896917701245

[20] Bennett JA , Braga AC, Pinto A, Van de Vijver K, Cornejo K, Pesci A, . Uterine PEComas: a morphologic, immunohistochemical, and molecular analysis of 32 tumors. Am J Surg Pathol 2018;42:1370–83. 30001237 10.1097/PAS.0000000000001119 PMC6133752

[21] Yang SR , Lin CY, Stehr H, Long SR, Kong CS, Berry GJ, . Comprehensive genomic profiling of malignant effusions in patients with metastatic lung adenocarcinoma. J Mol Diagn 2018;20:184–94. 29269277 10.1016/j.jmoldx.2017.10.007

[22] Testa S , Kumar J, Goodell AJ, Zehnder JL, Alexander KM, Sidana S, . Prevalence, mutational spectrum and clinical implications of clonal hematopoiesis of indeterminate potential in plasma cell dyscrasias. Semin Oncol 2022;49:465–75. 36503855 10.1053/j.seminoncol.2022.11.001

[23] Eisenhauer EA , Therasse P, Bogaerts J, Schwartz LH, Sargent D, Ford R, . New response evaluation criteria in solid tumours: revised RECIST guideline (version 1.1). Eur J Cancer 2009;45:228–47. 19097774 10.1016/j.ejca.2008.10.026

[24] Argani P , Aulmann S, Illei PB, Netto GJ, Ro J, Cho HY, . A distinctive subset of PEComas harbors TFE3 gene fusions. Am J Surg Pathol 2010;34:1395–406. 20871214 10.1097/PAS.0b013e3181f17ac0

[25] Malinowska I , Kwiatkowski DJ, Weiss S, Martignoni G, Netto G, Argani P. Perivascular epithelioid cell tumors (PEComas) harboring TFE3 gene rearrangements lack the TSC2 alterations characteristic of conventional PEComas: further evidence for a biological distinction. Am J Surg Pathol 2012;36:783–4. 22456611 10.1097/PAS.0b013e31824a8a37 PMC3327756

[26] McBride A , Garcia AJ, Sanders LJ, Yiu K, Cranmer LD, Kuo PH, . Sustained response to pembrolizumab in recurrent perivascular epithelioid cell tumor with elevated expression of programmed death ligand: a case report. J Med Case Rep 2021;15:400. 34301321 10.1186/s13256-021-02997-x PMC8305520

[27] Lattanzi M , Deng FM, Chiriboga LA, Femia AN, Meehan SA, Iyer G, . Durable response to anti-PD-1 immunotherapy in epithelioid angiomyolipoma: a report on the successful treatment of a rare malignancy. J Immunother Cancer 2018;6:97. 30285856 10.1186/s40425-018-0415-x PMC6167873

[28] Goodman AM , Kato S, Bazhenova L, Patel SP, Frampton GM, Miller V, . Tumor mutational burden as an independent predictor of response to immunotherapy in diverse cancers. Mol Cancer Ther 2017;16:2598–608. 28835386 10.1158/1535-7163.MCT-17-0386 PMC5670009

[29] Ready N , Hellmann MD, Awad MM, Otterson GA, Gutierrez M, Gainor JF, . First-line nivolumab plus ipilimumab in advanced non-small-cell lung cancer (CheckMate 568): outcomes by programmed death ligand 1 and tumor mutational burden as biomarkers. J Clin Oncol 2019;37:992–1000. 30785829 10.1200/JCO.18.01042 PMC6494267

[30] Gill S , Nowak AK, Bowyer S, Endersby R, Ebert MA, Cook A. Clinical evidence for synergy between immunotherapy and radiotherapy (SITAR). J Med Imaging Radiat Oncol 2022;66:881–95. 35699321 10.1111/1754-9485.13441 PMC9543060

[31] Wang Y , Deng W, Li N, Neri S, Sharma A, Jiang W, . Combining immunotherapy and radiotherapy for cancer treatment: current challenges and future directions. Front Pharmacol 2018;9:185. 29556198 10.3389/fphar.2018.00185 PMC5844965

[32] Shen Q , Rao Q, Xia QY, Yu B, Shi QL, Zhang RS, . Perivascular epithelioid cell tumor (PEComa) with TFE3 gene rearrangement: clinicopathological, immunohistochemical, and molecular features. Virchows Arch 2014;465:607–13. 25239799 10.1007/s00428-014-1655-x

[33] Agaram NP , Sung YS, Zhang L, Chen CL, Chen HW, Singer S, . Dichotomy of genetic abnormalities in PEComas with therapeutic implications. Am J Surg Pathol 2015;39:813–25. 25651471 10.1097/PAS.0000000000000389 PMC4431898

[34] Schmiester M , Dolnik A, Kornak U, Pfitzner B, Hummel M, Treue D, . TFE3 activation in a TSC1-altered malignant PEComa: challenging the dichotomy of the underlying pathogenic mechanisms. J Pathol Clin Res 2021;7:3–9. 33180365 10.1002/cjp2.187 PMC7737753

