# Supplementary material for: Systemic Treatments and Molecular Biomarkers for Perivascular Epithelioid Cell Tumors: A Single-institution Retrospective Analysis
Source: Cancer Res Commun. 2023 Jul 12;3(7):1212–23. doi: 10.1158/2767-9764.CRC-23-0139 (PMC10335919; doi:10.1158/2767-9764.CRC-23-0139)
Supplement: Figure S1 — Supplementary Figure S1 showing study flow diagram with details regarding the pathology cases review and patient selection process. [file crc-23-0139-s01.docx]

|  |
| --- |
| **Figure S1**. Study flow diagram showing pathology cases review process and patient selection. cPFS: clinical progression-free survival; OS: overall survival; OSH: outside hospital; PEComa: Perivascular epithelioid cell tumors; AML: angiomyolipoma; LAM: lymphangioleiomyomatosis; mTOR: mammalian target of rapamycin; ICI: Immune check-point inhibitors; RECIST 1.1.: Response Evaluation Criteria in Solid Tumors. Other: Olaparib (*n*=1); pazopanib (*n*=1), pazopanib-everolimus (*n*=1), anastrozole (*n*=1), and levantinib-everolimus (*n*=1). |
