# Supplementary material for: Systemic Treatments and Molecular Biomarkers for Perivascular Epithelioid Cell Tumors: A Single-institution Retrospective Analysis
Source: Cancer Res Commun. 2023 Jul 12;3(7):1212–23. doi: 10.1158/2767-9764.CRC-23-0139 (PMC10335919; doi:10.1158/2767-9764.CRC-23-0139)
Supplement: Figure S2 — shows an Oncoplot of the mutations discovered in patients for which next-generation sequencing testing was available. [file crc-23-0139-s02.docx]

|  |
| --- |
| **Figure S2**. **Oncoplot showing the mutations discovered in patients for which next-generation sequencing testing was available (*n*=12).** Each row represents a gene while each column represents a patient. The squares are color-coded to indicate the mutation type. For some patients a full genetic report with the specific mutation type classification was not available. The only patient with Angiomyolipoma in the graph had germline mutation testing rather than tumor testing, in contrast to all the other patients here represented. The bar graph on top shows the number of mutations per patient, using the same color-coding to represent the mutation type. The percentages on the right show the frequency of mutations in each gene out of all the mutations identified. PEComa: perivascular epithelioid cell tumors. |
