# Supplementary material for: Systemic Treatments and Molecular Biomarkers for Perivascular Epithelioid Cell Tumors: A Single-institution Retrospective Analysis
Source: Cancer Res Commun. 2023 Jul 12;3(7):1212–23. doi: 10.1158/2767-9764.CRC-23-0139 (PMC10335919; doi:10.1158/2767-9764.CRC-23-0139)
Supplement: Figure S3 — shows a bar graph with the number of treatment episodes in each disease group classified based on type and line of treatment. [file crc-23-0139-s03.docx]

|  |
| --- |
| **Figure S3**. **Bar graph showing the number of treatment episodes in each disease group classified based on type and line of treatment**. All the treatments here showed were administered in the adjuvant setting except one (shown with an asterisk). Other included: Olaparib (*n*=1) for second line in Malignant PEComa; pazopanib (*n*=1), pazopanib-everolimus (*n*=1), and anastrozole (*n*=1) for third line and above in Malignant PEComa; and levantinib-everolimus (*n*=1) in AML. ICI: Immune Checkpoint Inhibitors, including ipilimumab-nivolumab (*n*=1), and nivolumab single-agent (*n*=1) in AML, and pembrolizumab (*n*=1), and ipilimumab-nivolumab (*n*=1) as second line in Malignant PEComa. Anthracycline-based regimens included: vincristine-doxorubicin-cyclophosphamide, plus ifosfamide-etoposide (VAC-IE, *n*=1), ifosfamide-doxorubicin (*n*=1), and doxorubicin single-agent (*n*=1). LAM: lymphangioleiomyomatosis. |
