# Supplementary material for: Systemic Treatments and Molecular Biomarkers for Perivascular Epithelioid Cell Tumors: A Single-institution Retrospective Analysis
Source: Cancer Res Commun. 2023 Jul 12;3(7):1212–23. doi: 10.1158/2767-9764.CRC-23-0139 (PMC10335919; doi:10.1158/2767-9764.CRC-23-0139)
Supplement: Figure S5 — shows Kaplan-Meier Curves of Overall Survival for patients with Malignant PEComas treated with chemotherapy or mTOR inhibitors, and for patients with and without TP53 mutations, with and without TSC1/TSC2 mutations, and for those that are TFE3 positive versus TFE3 negative. [file crc-23-0139-s05.docx]

|  |
| --- |
| **Figure S5**. **Overall survival from first-line treatment in malignant PEComas**. **A**. Kaplan-Meier curve shows overall survival (OS) for patients treated with chemotherapy and those treated with mTOR inhibitors. **B**. Kaplan-Meier curve shows OS of patients with TFE3 positivity as detected through either IHC or FISH compared to those that were TFE3 negative. **C**. Kaplan-Meier curve shows OS in patients with malignant PEComa based on *TP53* mutational status. **D**. Kaplan-Meier curve shows OS in patients with PEComas based on *TSC1*/*TSC2* mutational status. Log-Rank *P*-values are shown. TP53_MUT: *TP53* mutated; TP53_WT: *TP53* wild-type; TSC1_MUT: *TSC1* mutated; TSC2_MUT: *TSC2* mutated; TSC1/TSC2_WT: *TSC1* or *TSC1* wild-type; TFE3_Pos: TFE3 positive; TFE3_Neg: TFE3 negative. |
