# Supplementary material for: Systemic Treatments and Molecular Biomarkers for Perivascular Epithelioid Cell Tumors: A Single-institution Retrospective Analysis
Source: Cancer Res Commun. 2023 Jul 12;3(7):1212–23. doi: 10.1158/2767-9764.CRC-23-0139 (PMC10335919; doi:10.1158/2767-9764.CRC-23-0139)
Supplement: Figure S6 — shows Kaplan-Meier curves for clinical PFS from first-line therapy in patients with PEComas, and specifically effect of TFE3 positivity, TSC1/TSC2 mutational status, and uterine versus extra-uterine location on clinical PFS. [file crc-23-0139-s06.docx]

|  |
| --- |
| **Figure S6**. **Clinical progression-free survival from first-line therapy in patients with PEComas**. **A**. Kaplan-Meier curve shows clinical progression-free survival (cPFS) for all the patients in the study. The halo around the curve represents the 95% confidence interval (CI). **B**. Kaplan-Meier curve shows cPFS in patients with PEComas based on TFE3 status. **C**. Kaplan-Meier curve shows cPFS in patients with PEComas based on *TSC1*/*TSC2* mutational status. **D**. Kaplan-Meier curve shows cPFS in patients with PEComas based on primary tumor site. Log-Rank *P*-values are shown. TFE3_Pos: TFE3 positive; TFE3_Neg: TFE3 negative; TSC1_MUT: *TSC1* mutated; TSC2_MUT: *TSC2* mutated; TSC1/TSC2_WT: *TSC1* or *TSC1* wild-type*.* NR: not reached. |
