# Supplementary material for: Systemic Treatments and Molecular Biomarkers for Perivascular Epithelioid Cell Tumors: A Single-institution Retrospective Analysis
Source: Cancer Res Commun. 2023 Jul 12;3(7):1212–23. doi: 10.1158/2767-9764.CRC-23-0139 (PMC10335919; doi:10.1158/2767-9764.CRC-23-0139)
Supplement: Figure S7 — shows Kaplan-Meier curves for combined PFS in patients with PEComas, specifically showing effect of TP53 and TSC1/TSC2 mutational status, as well as uterine vs extra-uterine location on combined PFS. [file crc-23-0139-s07.docx]

|  |
| --- |
| **Figure S7**. **Combined clinical progression-free survival in patients with PEComas in the whole cohort, including all treatment episodes regardless of line of therapy**. **A**. Kaplan-Meier curve shows clinical progression-free survival (cPFS) for all treatment episodes and for all the patients in the study. The halo around the curve represents the 95% confidence interval (CI). **B**. Kaplan-Meier curve shows cPFS in patients with PEComas based on *TP53* mutational status and including all treatment episodes. **C**. Kaplan-Meier curve shows cPFS in patients with PEComas based on *TSC1*/*TSC2* mutational status and including all treatment episodes. **D**. Kaplan-Meier curve shows cPFS in patients with PEComas based on primary tumor site and including all treatment episodes. Log-Rank *P*-values are shown. TP53_MUT: *TP53* mutated; TP53_WT: *TP53* wild-type; TSC1_MUT: *TSC1* mutated; TSC2_MUT: *TSC2* mutated; TSC1/TSC2_WT: *TSC1* or *TSC1* wild-type*.* NR: not reached. |
