# Supplementary material for: Systemic Treatments and Molecular Biomarkers for Perivascular Epithelioid Cell Tumors: A Single-institution Retrospective Analysis
Source: Cancer Res Commun. 2023 Jul 12;3(7):1212–23. doi: 10.1158/2767-9764.CRC-23-0139 (PMC10335919; doi:10.1158/2767-9764.CRC-23-0139)
Supplement: Figure S8 — shows combined clinical progression-free survival (cPFS) in the whole cohort comparing each mTOR inhibitors to other treatments. [file crc-23-0139-s08.docx]

|  |
| --- |
| **Figure S8**. **Combined clinical progression-free survival (cPFS) comparing each mTOR inhibitors to other treatments in the whole cohort, including all treatment episodes regardless of line of therapy**. Kaplan-Meier curve shows combined cPFS for the whole patient cohort, comparing everolimus, nab-sirolimus, temsirolimus, sirolimus, chemotherapy, immune checkpoint inhibitors (ICI), and other treatments including olaparib (*n*=1), pazopanib (*n*=1), pazopanib-everolimus (*n*=1), anastrozole (*n*=1), and levantinib-everolimus (*n*=1). CI: confidence interval; HR: hazard ratio; NR: not reached. Shown in the table are the Cox-Wald *P*-values and the HR obtained from a multivariable Cox proportional hazard analysis including the treatment variable (everolimus, nab-sirolimus, sirolimus, temsirolimus, ICI, other, chemotherapy), together with the following variables: adjuvant treatment, histology, number of lines of therapy, age at diagnosis, and the frailty covariate. |
