# Supplementary material for: Systemic Treatments and Molecular Biomarkers for Perivascular Epithelioid Cell Tumors: A Single-institution Retrospective Analysis
Source: Cancer Res Commun. 2023 Jul 12;3(7):1212–23. doi: 10.1158/2767-9764.CRC-23-0139 (PMC10335919; doi:10.1158/2767-9764.CRC-23-0139)
Supplement: Figure S9 — shows Kaplan-Meier curves for combined clinical PFS in patients with malignant PEComas. Specifically, figure shows effects on combined clinical PFS of TFE3 positivity, treatment type, and TP53 and TSC1/TSC2 mutational status. [file crc-23-0139-s09.docx]

|  |
| --- |
| **Figure S9**. **Combined clinical progression-free survival in patients with Malignant PEComa including all treatment episodes, regardless of line of therapy**. **A**. Kaplan-Meier curve shows clinical progression-free survival (cPFS) for patients treated with ICI, mTOR inhibitors, chemotherapy and other treatments. **B**. Kaplan-Meier curve shows cPFS in patients with TFE3 positivity as detected through either IHC or FISH compared to those that were TFE3 negative. **C**. Kaplan-Meier curve shows cPFS in patients with PEComas based on TP53 mutational status. **D**. Kaplan-Meier curve shows cPFS in patients with PEComas based on *TSC1*/*TSC2* mutational status. Log-Rank *P*-values are shown. TP53_MUT: *TP53* mutated; TP53_WT: *TP53* wild-type; TSC1_MUT: *TSC1* mutated; TSC2_MUT: *TSC2* mutated; TSC1/TSC2_WT: *TSC1* or *TSC1* wild-type*.* TFE3_Neg: TFE3 negative; TFE3_Pos: TFE3 positive; NR: not reached. |
