# Supplementary material for: Systemic Treatments and Molecular Biomarkers for Perivascular Epithelioid Cell Tumors: A Single-institution Retrospective Analysis
Source: Cancer Res Commun. 2023 Jul 12;3(7):1212–23. doi: 10.1158/2767-9764.CRC-23-0139 (PMC10335919; doi:10.1158/2767-9764.CRC-23-0139)
Supplement: Figure S10 — shows a Kaplan-Meier curve that represents effects of different treatments on combined clinical PFS only in patients with malignant PEComa. [file crc-23-0139-s10.docx]

|  |
| --- |
| **Figure S10**. **Combined clinical progression-free survival (cPFS) in malignant PEComa only**. Kaplan-Meier curve shows combined cPFS only in patients with malignant PEComa, comparing everolimus, nab-sirolimus, temsirolimus, sirolimus, chemotherapy, immune checkpoint inhibitors (ICI), and other treatments including olaparib (*n*=1), pazopanib (*n*=1), pazopanib-everolimus (*n*=1), and anastrozole (*n*=1). CI: confidence interval; HR: hazard ratio; NR: not reached. Shown in the table are the Cox-Wald *P*-values and the HR obtained from a multivariable Cox proportional hazard analysis including the treatment variable, the adjuvant treatment variable, and the frailty covariate. |
