# Supplementary material for: Systemic Treatments and Molecular Biomarkers for Perivascular Epithelioid Cell Tumors: A Single-institution Retrospective Analysis
Source: Cancer Res Commun. 2023 Jul 12;3(7):1212–23. doi: 10.1158/2767-9764.CRC-23-0139 (PMC10335919; doi:10.1158/2767-9764.CRC-23-0139)
Supplement: Table S1 — shows a a description of study design, main findings and conclusions for the most relevant references in the study regarding management of patients with PEComas. [file crc-23-0139-s11.docx]

| **Table S1. Management of Perivascular Epithelioid Cell Tumors** | | | |
| --- | --- | --- | --- |
| **Reference** | **Study Design** | **Results** | **Comments/Conclusions** |
| Italiano et al^9^ | - Report of 2 cases of patients with malignant PEComa treated with temsirolimus | - First case was a 69 years-old woman with malignant PEComa of the uterus metastatic to the lungs that showed a 35% reduction in metastatic tumor burden one month after starting temsirolimus - Second case was a 55 years-old woman with malignant PEComa of the uterus with metastatic disease in the lungs and liver, relapsed after cytotoxic chemotherapy, which showed a partial response at 8 months after administration of temsirolimus | - First report of clinical activity of the mTOR inhibitor temsirolimus in patients with malignant PEComa not associated with the tuberous sclerosis complex |
| Benson et al.^10^ | - Retrospective study of 10 patients with malignant PEComa receiving sirolimus (*n*=9) or temsirolimus (*n*=1) | - 5 patients showed partial response, 1 patient showed stable disease, and another patient showed progressive disease based on RECIST 1.1 - 2 patients progressed rapidly and were not assessable for response - 1 patient stopped the treatment early due to grade 3 hyperlipidemia - At a median follow-up of 1.9 years, median OS was 2.4 years (95% IC: 0.3-4.5), with a 1-year survival rate of 78.8%. | - First case series of patients with malignant PEComa receiving mTOR inhibitors with good radiographic response, and good safety profile |
| Wagner et al.^11^ | - Retrospective analysis of 3 patients with metastatic and/or recurrent malignant PEComa treated with sirolimus | - One patient exhibited a near complete response that had lasted for 16 months at last follow-up - One patient showed 40% reduction in largest tumor diameter at 9 months with disease control lasting for 10 months - Disease stabilization in one patient where sirolimus was administered with sorafenib 200 mg twice daily - All tumors showed overexpression of phosphorylated S6 protein, marker of mTORC1 activation - All tumors showed reduction in expression of tuberin, product of *TSC2* - Only one tumor showed biallelic deletion of *TSC1* | - First clinical report of activity of sirolimus in patients with malignant PEComa outside of the tuberous sclerosis complex - Correlative studies showed that overactivation of mTORC1 through loss of function of the TSC1/TSC2 repressor complex might be an important event in the pathogenesis of sporadic, non-tuberous sclerosis complex-associated, PEComas |
| Dikson et al.^12^ | - Retrospective review of 5 patients with extrarenal non-pulmonary PEComas treated with either sirolimus or everolimus | - The authors reported 3 complete responses, 1 partial response and 1 case of progressive disease - *TSC2* mutations were found in 4/5 patients and no *TFE3* translocation or TFE3 protein overexpression were noted in the patient that had progressive disease | - Loss of function mutations in *TSC2* were found in 4/5 patients - Aggregated analysis with prior studies of *TSCI*/*TSC2* mutations in PEComas showed loss of function events in 85% of cases, which overall correlated with overexpression of pS6 on IHC - CR was observed In 80% of patients with either *TSCI*/*TSC2* loss or pS6 overexpression |
| Weeber et al.^13^ | - Single case report of a patient with metastatic malignant PEComa treated with everolimus | - The patient experienced a complete response after starting everolimus that lasted approximately 3 years - IHC showed overexpression of phosphorylated S6 and mTOR, indicative of mTOR pathway activation - No mutations in *TSC1*/*TSC2* were noted - Array CGH analysis showed significant aneuploidy | - mTOR inhibitors like everolimus can achieve complete responses in metastatic malignant PEComa - Correlative studies showed that the molecular mechanisms leading to overactivation of the mTOR pathway might be more complex than simple loss of function mutations in *TSC1*/*TSC2* |
| Bissler et al.^14^ | - 24-month long nonrandomized, open-label, phase 1/2 clinical trial of sirolimus in patients with AML in the context of the tuberous sclerosis complex or of sporadic LAM - Sirolimus was administered for the first 12 months only, followed by 12 months of observation with serial imaging - Primary endpoint was AML volume reduction | - A total of 25 patients were enrolled, with 20 patients completing the 12-month evaluation and 18 completing the 24-month evaluation - The mean AML volume at 12 months reduced at 53.2% of the baseline value with an increase up to 85.9% of the baseline value during the following 12 months off sirolimus - Among patients with concomitant pulmonary LAM, there was an increase in forced vital capacity while on sirolimus that was reversed during the 12 months off sirolimus - Serious adverse events were observed in 5 patients and included: diarrhea, pyelonephritis, stomatitis, and respiratory infections | - Treatment with sirolimus reduced AML volume and improved spirometric measurements for patients with concomitant pulmonary LAM - These effects were partially reversed when sirolimus was stopped |
| Bissler et al.^15^ | - Phase 3, multicentric, placebo-controlled, randomized, double-blind trial studying everolimus in patients 18 years or older with at least one AML in the context of either the tuberous sclerosis complex or sporadic LAM - The primary efficacy endpoint was the proportion of patients with at least a 50% reduction in AML volume compared to baseline | - A total of 118 patients were enrolled, and 79 were assigned to the everolimus arm, while 39 were assigned to the placebo arm - The AML response rate was 42% for the everolimus arm and 0% for the placebo arm - Therapy was discontinued due to disease progression in 9 placebo patients, and due to adverse events in 4 placebo patients and 2 everolimus patients - Most common side effects included stomatitis, nasopharyngitis, and acneic skin lesions | - Everolimus effectively reduced AML volume in patients with sporadic LAM or tuberous sclerosis complex with a good safety profile |
| Wagner et al.^16^ | - Phase 2, single-arm, perspective clinical trial of nab-sirolimus in patient with malignant PEComa - Nab-sirolimus was administered intravenously at a dose of 100 mg/m^2^ one weekly for 2 weeks in 3-week cycles - Primary endpoint was objective response rate - Secondary endpoints included duration of response, PFS, safety, and tumor biomarker analysis | - 35 patients were enrolled, 34 were evaluable for safety and 31 were assessable for efficacy - The overall response rate was 39%, with one complete response and 11 partial responses - Stable disease was observed in 52% of patients, while 10% of patients had progressive disease - 89% of patients with a *TSC2* mutation achieved a response compared to 13% only of patients without a *TSC2* mutation - Median PFS was 10.6 months while median OS was 40.8 months - The most common adverse events were mucositis, rash, and fatigue, and these were mostly of grade 1 or 2 | - Nab-sirolimus is a safe and effective treatment option for patients with malignant PEComa - Mutations in *TSC2* strongly correlated with response to nab-sirolimus, while absence of phosphorylated S6 staining on IHC correlated with lack of response to nab-sirolimus |
| Sanfilippo et al.^17^ | - Observational, retrospective, multicentric study of patients with advanced/metastatic PEComa treated with systemic therapy - Endpoints measured included OS, PFS, ORR, and DCR | - A total of 53 patients were included in the study - Gemcitabine-based chemotherapy regimens showed a median PFS of 3.4 months with an ORR of 20%, similar to anthracycline-based regimens (median PFS: 3.2 months, and ORR of 13%) - Antiangiogenic agents showed an ORR of 8.3% and a median PFS of 5.4 months, with most patients achieving disease stabilization - mTOR inhibitors including everolimus, temsirolimus and sirolimus showed an ORR of 41% and a median PFS of 9 months | - Overall, this study suggests that mTOR inhibitors are the most effective agents in the treatment of advanced/metastatic PEComa - Antiangiogenic agents and cytotoxic chemotherapy showed overall worse response rates and shorted survival compared to mTOR inhibitors |
| Lee et al.^18^ | - Case report of a patient with epithelioid AML metastatic to the lung that responded to sorafenib | - The authors report the case of a 30 years-old male with renal epithelioid AML localized at diagnosis, that was initially resected with metastatic recurrence to the lungs four years after removal of the primary tumor - The patient was started on sorafenib administered orally at the dose of 400 mg twice daily with radiographic response of the main pulmonary mass - The patient underwent pulmonary metastectomy and remained with no evidence of disease at last follow-up | - First report of activity of sorafenib in patients with PEComas |
| Machado et al.^19^ | - Case report of a patient with metastatic malignant PEComa refractory to several cytotoxic chemotherapy regimens | - The authors report a case of a 33 years-old female with malignant PEComa of the omentum metastatic to the liver at diagnosis - The patients received doxorubicin as first-line with disease progression, followed by ifosfamide again with disease progression - Disease stabilization for 5 months was obtained with temsirolimus administered as third-line - Further lines of therapy included trabectedin, docetaxel/gemcitabine, and dacarbazine-based regimens with no response - The patient died 30 months after the initial diagnosis | - Case report showing poor response of malignant PEComas to cytotoxic chemotherapy |
| McBride et al.^25^ | - Case report of a patient with metastatic malignant PEComa expressing PD-1 | - The authors report on the case of a 69 years-old Caucasian female with malignant PEComa of the abdominal wall initially treated with surgery and chemotherapy, which showed metastatic recurrence to the lungs - The tumor expressed high levels of PD-1 on IHC and the patients was started on pembrolizumab at recurrence, achieving a complete response after 21 cycles - The patients was treated with a total of 40 cycles and remained disease-free at last follow-up, 19 months after the last treatment cycle | - Case report showing efficacy of immune checkpoint inhibitors in PEComas that express high levels of PD-1 |
