# Supplementary material for: Systemic Treatments and Molecular Biomarkers for Perivascular Epithelioid Cell Tumors: A Single-institution Retrospective Analysis
Source: Cancer Res Commun. 2023 Jul 12;3(7):1212–23. doi: 10.1158/2767-9764.CRC-23-0139 (PMC10335919; doi:10.1158/2767-9764.CRC-23-0139)
Supplement: Table S2 — shows the results of molecular testing available for each patient in the study. [file crc-23-0139-s12.docx]

**Table S2**. Molecular testing results for each patient in the study.

| **Patients** | **Histology** | **NGS** | **FISH** | **IHC** | **TFE3** | **TSC1/TSC2** | **TP53** |
| --- | --- | --- | --- | --- | --- | --- | --- |
| 1 | Malignant PEComa | Yes – TFE3 fusion positive | NA | NA | positive NGS | wt | wt |
| 2 | Malignant PEComa | Yes – TFE3 fusion positive | TFE3 positive | TFE3  positive | positive FISH/NGS/IHC | wt | wt |
| 3 | Malignant PEComa | Yes - no fusion testing | TFE3 positive | NA | positive FISH | TSC1 mut | mutated |
| 4 | Malignant PEComa | Yes - no fusion testing | TFE3 positive | TFE3  positive | positive FISH/IHC | wt | wt |
| 5 | Malignant PEComa | Yes - no fusion testing | NA | TFE3  positive | positive IHC | TSC2 mut | mutated |
| 6 | Malignant PEComa | Yes - no fusion testing | NA | TFE3  positive | positive IHC | TSC2 mut | mutated |
| 7 | Malignant PEComa | Yes - TFE3 fusion negative | NA | TFE3  negative | negative NGS/IHC | TSC1 mut | wt |
| 8 | Malignant PEComa | Yes - TFE3 fusion negative | NA | NA | negative NGS | TSC1 mut | wt |
| 9 | Malignant PEComa | NA | NA | TFE3  negative | negative IHC | NA | NA |
| 10 | Malignant PEComa | NA | NA | NA | negative | NA | NA |
| 11 | Malignant PEComa | Yes - no fusion testing | NA | NA | negative | TSC2 mut | wt |
| 12 | Malignant PEComa | Yes - no fusion testing | NA | NA | negative | TSC2 mut | mutated |
| 13 | Malignant PEComa | NA | NA | NA | negative | NA | NA |
| 14 | Malignant PEComa | NA | NA | NA | negative | NA | NA |
| 15 | Malignant PEComa | NA | NA | NA | negative | NA | NA |
| 16 | Malignant PEComa | NA | NA | NA | negative | NA | NA |
| 17 | Malignant PEComa | Yes - no fusion testing | NA | NA | negative | TSC1 mut | mutated |
| 18 | LAM | NA | NA | NA | negative | NA | NA |
| 19 | LAM | NA | NA | NA | negative | NA | NA |
| 20 | LAM | NA | NA | NA | negative | NA | NA |
| 21 | AML | NA | NA | NA | negative | TSC2 (GL) | NA |
| 22 | Epithelioid AML | NA | NA | NA | negative | NA | NA |
| 23 | Epithelioid AML | NA | NA | NA | negative | NA | NA |
| 24 | Epithelioid AML | NA | NA | NA | negative | NA | NA |
| 25 | AML | NA | NA | NA | negative | NA | NA |
| 26 | Epithelioid AML | NA | NA | NA | negative | NA | NA |
| 27 | Epithelioid AML | NA | NA | NA | negative | NA | NA |
| 28 | AML | NA | NA | NA | negative | NA | NA |
| 29 | AML | NA | NA | NA | negative | NA | NA |

NA: not available; IHC: immunohistochemistry; NGS: next generation sequencing; FISH: fluorescence in-situ hybridization; TSC1 mut: TSC1 mutated; TSC2 mut: TSC2 mutated; wt: wild type; TSC2 (GL): TSC2 germ-line mutation.
