# Supplementary material for: Systemic Treatments and Molecular Biomarkers for Perivascular Epithelioid Cell Tumors: A Single-institution Retrospective Analysis
Source: Cancer Res Commun. 2023 Jul 12;3(7):1212–23. doi: 10.1158/2767-9764.CRC-23-0139 (PMC10335919; doi:10.1158/2767-9764.CRC-23-0139)
Supplement: Table S3 — shows the best overall response rate (ORR) and disease control rate (DCR) for different treatment types based on histology, tumor location, TFE3 positivity, as well as TSC1, TSC2, and TP53 mutational status. [file crc-23-0139-s13.docx]

**Table S3**. Best overall response rate (ORR) and disease control rate (DCR) for different treatment types based on histology, tumor location, TFE3 positivity, as well as *TSC1*, *TSC2*, and *TP53* mutational status.

| **Treatment** |  | **All Patients** | **Malignant PEComa** | **Epithelioid AML/AML** | **LAM** | ***TSC1* mut** | ***TSC2* mut** | ***TP53* mut** | **Uterine** | **TFE3 positive** |
| --- | --- | --- | --- | --- | --- | --- | --- | --- | --- | --- |
| mTOR inhibitors | *N* (treatment episodes) | 36 | 22 | 11 | 3 | 3 | 7 | 7 | 12 | 7 |
|  | Best ORR, *N* (%) | 6(16.7) | 3(13.6) | 2(18.2) | 1(33.3) | 1(33.3) | 1(14.3) | 1(14.3) | 2(16.7) | 1(14.0) |
|  | Best DCR, *N* (%) | 28(77.8) | 14(63.6) | 11(100) | 3(100) | 3(100) | 5(71.4) | 6(85.7) | 6(50.0) | 5(71.0) |
| Cytotoxic Chemotherapy | *N* (treatment episodes) | 6 | 6 | 0 | 0 | 4 | 0 | 0 | 4 | 1 |
|  | Best ORR, *N* (%) | 0(0) | 0(0) | – | – | 0 | – | – | 0 | 0 |
|  | Best DCR, *N* (%) | 5(83.3) | 5(83.3) | – | – | 3(75.0) | – | – | 3(75.0) | 1(100) |
| ICI | *N* (treatment episodes) | 3 | 3 | 0 | 0 | 2 | 0 | 2 | 2 | 2 |
|  | Best ORR, *N* (%) | 2(66.7) | 2(66.7) | – | – | 1(50.0) | – | 1(50.0) | 1(50.0) | 1(50.0) |
|  | Best DCR, *N* (%) | 2(66.7) | 2(66.7) | – | – | 1(50.0) | – | 1(50.0) | 1(50.0) | 1(50.0) |
| Temsirolimus | *N* (treatment episodes) | 5 | 4 | 1 | 0 | 0 | 1 | 1 | 3 | 1 |
|  | Best ORR, *N* (%) | 2(40.0) | 1(25.0) | 1(100) | – | – | 0 | 0 | 1(33.3) | 0 |
|  | Best DCR, *N* (%) | 3(60.0) | 2(50.0) | 1(100) | – | – | 0 | 0 | 0 | 0 |
| Everolimus | *N* (treatment episodes) | 13 | 7 | 6 | 0 | 0 | 3 | 1 | 3 | 0 |
|  | Best ORR, *N* (%) | 1(13.3) | 0 | 1(16.7) | – | – | 0 | 0 | 0 | – |
|  | Best DCR, *N* (%) | 10(76.9) | 4(57.1) | 6(100) | – | – | 2(66.6) | 1(100) | 1(33.3) | – |
| Sirolimus | *N* (treatment episodes) | 11 | 4 | 4 | 3 | 0 | 2 | 2 | 2 | 3 |
|  | Best ORR, *N* (%) | 2(18.2) | 1(25.0) | 0 | 1(33.3) | – | 1(50.0) | 1(50.0) | 0 | 1(33.3) |
|  | Best DCR, *N* (%) | 10(90.9) | 3(75.0) | 4(100) | 3(100) | – | 2(100) | 2(100) | 1(50.0) | 3(100) |
| Nab-sirolimus | *N* (treatment episodes) | 7 | 7 | 0 | 0 | 3 | 1 | 3 | 4 | 3 |
|  | Best ORR, *N* (%) | 1(14.3) | 1(14.3) | – | – | 1(33.3) | 0 | 0 | 1(25.0) | 0 |
|  | Best DCR, *N* (%) | 5(71.4) | 5(71.4) | – | – | 3(100) | 1(100) | 3(100) | 3(75.0) | 2(66.7) |
| Other*^a^* | *N* (treatment episodes) | 4 | 3 | 1 | 0 | 1 | 0 | 0 | 1 | 2 |
|  | Best ORR, *N* (%) | 0 | 0 | 0 | – | 0 | – | – | 0 | 0 |
|  | Best DCR, *N* (%) | 2(50.0) | 1(33.3) | 1(100) | – | 0 | – | – | 0 | 1(50.0) |

ORR: overall response rate; DCR: disease control rate; mTOR: mammalian target of rapamycin; PEComa: perivascular epithelioid cell tumors; AML: angiomyolipoma; LAM: lymphangioleiomyomatosis; Other*^a^*: everolimus-levantinib (*n*=1), anastrozole (*n*=1), olaparib (*n*=1), pazopanib-everolimus (*n*=1); ICI: immune checkpoint inhibitors.
