# Supplementary material for: Systemic Treatments and Molecular Biomarkers for Perivascular Epithelioid Cell Tumors: A Single-institution Retrospective Analysis
Source: Cancer Res Commun. 2023 Jul 12;3(7):1212–23. doi: 10.1158/2767-9764.CRC-23-0139 (PMC10335919; doi:10.1158/2767-9764.CRC-23-0139)
Supplement: Table S4 — shows the best disease control rate (DCR) and overall response rate (ORR) for mTOR inhibitors compared to other treatments based on histotype. [file crc-23-0139-s14.docx]

**Table S4**. Best disease control rate (DCR) and overall response rate (ORR) for mTOR inhibitors compared to other treatments based on histotype.

|  |  | **Best DCR (95% CI)** |  |  | **Best ORR (95% CI)** |  |
| --- | --- | --- | --- | --- | --- | --- |
| **Groups (*N* episodes)** | **mTOR Inhibitors** | **Other*^a^*** | ***P*-value**  **(Fisher’s)** | **mTOR Inhibitors** | **Other*^a^*** | ***P*-value**  **(Fisher’s)** |
| All Patients (*N*=49) | 77.8(61.9–88.3) | 69.2(42.4–87.3) | 0.70 | 16.7(7.9–31.9) | 15.4(2.7–42.2) | 0.99 |
| Malignant PEComa (*N*=34) | 63.3(42.9–80.3) | 66.7(39.1–86.2) | 0.99 | 13.6(4.7–33.3) | 16.7(2.9–44.8) | 0.99 |
| Epithelioid AML (*N*=15)  /AML/LAM | 100(78.5–100) | 100(5.1–100) | 0.99 | 21.4(7.5–47.6) | 0.0(0.0–94.9) | 0.99 |
|  |  |  |  |  |  |  |
|  | **Nab-Sirolimus** | **Everolimus/Sirolimus/**  **Temsirolimus** |  | **Nab-Sirolimus** | **Everolimus/Sirolimus/**  **Temsirolimus** |  |
| Malignant PEComa (*N*=22) | 71.4(35.8–94.9) | 60.0(35.7–80.2) | 0.99 | 14.3(0.7–51.3) | 13.3(2.4–37.9) | 0.99 |

ORR: overall response rate; DCR: disease control rate; mTOR: mammalian target of rapamycin; PEComa: perivascular epithelioid cell tumors; AML: angiomyolipoma; LAM: lymphangioleiomyomatosis; Other*^a^*: everolimus-levantinib (*n*=1), anastrozole (*n*=1), olaparib (*n*=1), pazopanib-everolimus (*n*=1), cytotoxic chemotherapy (*n*=6), immune checkpoint inhibitors (*n*=3). *P*-values were calculated with the Fisher’s exact test.
