# Supplementary material for: Systemic Treatments and Molecular Biomarkers for Perivascular Epithelioid Cell Tumors: A Single-institution Retrospective Analysis
Source: Cancer Res Commun. 2023 Jul 12;3(7):1212–23. doi: 10.1158/2767-9764.CRC-23-0139 (PMC10335919; doi:10.1158/2767-9764.CRC-23-0139)
Supplement: Table S5 — shows DCR and ORR based on biomarker status and primary tumor site, considering all treatment episodes, and mTOR inhibitors only. [file crc-23-0139-s15.docx]

**Table S5**. DCR and ORR based on biomarker status and primary tumor site, considering all treatment episodes, and mTOR inhibitors only.

| **Variables** | **All Treatments**  **Best DCR (95% CI)** | ***P*-value**  **(Fisher’s)** | **mTOR inhibitors**  **Best DCR (95% CI)** | ***P*-value**  **(Fisher’s)** | **All Treatments**  **Best ORR (95% CI)** | ***P*-value**  **(Fisher’s)** | **mTOR inhibitors**  **Best ORR (95% CI)** | ***P*-value**  **(Fisher’s)** |
| --- | --- | --- | --- | --- | --- | --- | --- | --- |
| **TFE3** |  |  |  |  |  |  |  |  |
| Positive | 66.7(39.1–86.2) | 0.45 | 71.4(35.9–94.9) | 0.63 | 16.7(2.9–44.8) | 0.99 | 14.3(0.7–51.3) | 0.99 |
| Negative | 78.4 (62.8–88.6) |  | 79.3(61.6–90.2) |  | 16.2(7.6–31.1) |  | 17.2(34.6–7.6) |  |
| ***TP53* Status** |  |  |  |  |  |  |  |  |
| Mutated | 77.8(45.3–96.0) | 0.99 | 85.7(46.7–99.3) | 0.99 | 22.2(3.9–54.7) | 0.63 | 14.3(0.7–51.3) | 0.99 |
| Wild Type | 75.0 (85.8–59.8) |  | 75.8(57.9–87.8) |  | 15.0(29.1–7.1) |  | 17.2(34.6–7.6) |  |
| ***TSC1*/*TSC2* Status** |  |  |  |  |  |  |  |  |
| Mutated | 70.6(46.9–86.7) | 0.72 | 80.0(49.0–96.4) | 0.99 | 17.6(6.2–41.0) | 0.99 | 20.0(3.5–50.9) | 0.99 |
| Wild Type | 78.1(61.2–88.9) |  | 76.9(57.9–88.9) |  | 15.6(6.8–31.7) |  | 15.4(6.1–33.5) |  |
| ***TSC2* Status** |  |  |  |  |  |  |  |  |
| Mutated | 71.4(35.8–94.9) | 0.99 | 71.4(35.8–94.9) | 0.64 | 14.3(0.7–51.3) | 0.99 | 14.3(0.7–51.3) | 0.99 |
| Wild Type | 76.2(61.4–86.5) |  | 79.3(61.6–90.2) |  | 16.7(8.3–30.6) |  | 17.2(7.6–34.5) |  |
| **Primary Site** |  |  |  |  |  |  |  |  |
| Uterine | 50.0(29.0–70.9) | **0.004** | 50.0(25.4–74.6) | **0.009** | 16.7(5.8–39.2) | 0.99 | 16.7(2.9–44.8) | 0.99 |
| Extra-uterine | 90.3(75.1–96.7) |  | 91.7(74.1–98.5) |  | 19.3(9.2–36.3) |  | 16.7(6.7–35.8) |  |
| **Primary Site (Malignant PEComa only)** |  |  |  |  |  |  |  |  |
| Uterine | 47.3(27.3–68.3) | 0.07 | 50.0(25.4–74.6) | 0.20 | 16.7(2.9–44.8) | 0.99 | 15.8(5.5–37.6) | 0.99 |
| Extra-uterine | 80.0(54.8–92.9) |  | 80.0(49.0–96.4) |  | 10.0(0.5–40.4) |  | 13.3(2.4–37.8) |  |

ORR: overall response rate; DCR: disease control rate; mTOR: mammalian target of rapamycin. PEComa: perivascular epithelioid cell tumors*;* 95% CI: 95% confidence interval*. P*-values were calculated with the Fisher’s exact test.
