# Supplementary material for: Systemic Treatments and Molecular Biomarkers for Perivascular Epithelioid Cell Tumors: A Single-institution Retrospective Analysis
Source: Cancer Res Commun. 2023 Jul 12;3(7):1212–23. doi: 10.1158/2767-9764.CRC-23-0139 (PMC10335919; doi:10.1158/2767-9764.CRC-23-0139)
Supplement: Table S6 — shows median Overall Survival in months, as well as 5-year Overall Survival rate for the whole cohort. [file crc-23-0139-s16.docx]

**Table S6A.** Median Overall Survival for the whole cohort.

|  | **Patients  (*N*)** | **Events (*N*)** | **Median (months)** | **95% CI (months)** |
| --- | --- | --- | --- | --- |
| TFE3 positive | 6 | 4 | 28.4 | (19.3–NR) |
| TFE3 negative | 23 | 6 | 210.6 | (210.6–NR) |
| *TP53*_WT | 24 | 7 | 210.6 | (210.6–NR) |
| *TP53*_MUT | 5 | 3 | 80.8 | (80.8–NR) |
| *TSC1*/*TSC2*_WT | 20 | 7 | 210.6071 | (81.6–NR) |
| *TSC1*_MUT | 4 | 1 | NR | (16.9–NR) |
| *TSC2*_MUT | 5 | 2 | 142.8214 | (80.8–NR) |
| Uterine | 9 | 6 | 204.8571 | (81.6–NR) |
| Extra-uterine | 20 | 4 | 210.6071 | (80.8–NR) |
| Malignant PEComa | 17 | 8 | 81.6 | (80.8–NR) |
| LAM/AML/Epithelioid AML | 12 | 2 | 216.5 | (NA) |
| mTOR inhibitors | 24 | 7 | 216.5 | (80.8–NR) |
| Chemotherapy | 5 | 3 | 204.9 | (81.6–NR) |
| Metastatic at diagnosis | 7 | 3 | 41.1 | (19.3–NR) |
| Localized at diagnosis | 22 | 7 | 210.6 | (204.9–NR) |

**Table S6B.** 5-year Overall Survival rate for the whole cohort.

|  | ***N* at risk** | **Events (*N*)** | **5-year OS rate (%)** | **95% CI**  **(%)** |
| --- | --- | --- | --- | --- |
| TFE3 positive | 2 | 1 | 31.2% | (6.7–100.0) |
| TFE3 negative | 18 | 1 | 89.5% | (76.7–100.0) |
| *TP53*_WT | 17 | 1 | 80.6% | (65.2–99.7) |
| *TP53*_MUT | 5 | 1 | 80.0% | (51.6–100.0) |
| *TSC1*/*TSC2*_WT | 15 | 1 | 78.5% | (61.8–99.7) |
| *TSC1*_MUT | 3 | 1 | 66.7% | (30.0–100.0) |
| *TSC2*_MUT | 5 | 0 | 100.0% | (NA) |
| Uterine | 4 | 1 | 56.2% | (28.1–100.0) |
| Extra-uterine | 7 | 1 | 69.9% | (47.8–100.0) |
| Malignant PEComa | 10 | 1 | 71.2% | (50.8–99.9) |
| LAM/AML/Epithelioid AML | 10 | 1 | 90.0% | (73.2–100.0) |
| mTOR inhibitors | 14 | 1 | 74.0% | (56.6–96.7) |
| Chemotherapy | 5 | 0 | 100.0% | (NA) |
| Metastatic at diagnosis | 7 | 1 | 47.6% | (18.8–100.0) |
| Localized at diagnosis | 19 | 1 | 89.2% | (76.0–100.0) |

NR: not reached; NA: not available. PEComa: perivascular epithelioid cell tumors; LAM: lymphangioleiomyomatosis; AML: angiomyolipoma; LAM: lymphangioleiomyomatosis; WT: wild-type; MUT: mutated; mTOR: mammalian target of rapamycin.
