# Supplementary material for: Systemic Treatments and Molecular Biomarkers for Perivascular Epithelioid Cell Tumors: A Single-institution Retrospective Analysis
Source: Cancer Res Commun. 2023 Jul 12;3(7):1212–23. doi: 10.1158/2767-9764.CRC-23-0139 (PMC10335919; doi:10.1158/2767-9764.CRC-23-0139)
Supplement: Table S7 — shows results of Cox proportional hazard analysis for overall survival in patients with malignant PEComa only. [file crc-23-0139-s17.docx]

**Table S7**. Cox proportional hazard analysis for overall survival in patients with malignant PEComa only.

| **Variables** | ***N* Episodes (%)** | **Univariable** | | | | **Multivariable** | |
| --- | --- | --- | --- | --- | --- | --- | --- |
|  |  | **Hazard Ratio**  **(95%CI)** | ***P*-value**  **(Cox-Wald)** | ***P*-value**  **(Log-Rank)** | | **Hazard Ratio**  **(95%CI)** | ***P*-value**  **(Cox-Wald)** |
| **Sex** |  |  |  | |  |  |  |
| Male | 4(23.5) | 0.2 (0.01–1.6) | 0.3 | | 0.12 |  |  |
| Female | 13(76.5) | **–** | **–** | |  |  |  |
| **Primary Site** |  |  |  | |  |  |  |
| Uterine | 9(52.9) | 1.1(0.2–4.9) | 0.9 | | 0.95 |  |  |
| Extra-uterine | 8(47.1) | **–** | **–** | |  |  |  |
| **Age, years (median, range)** | 48(5–71) | 1.0(0.9–1.1) | 0.1 | | 0.20 |  |  |
| **Metastatic at diagnosis** |  |  |  | |  |  |  |
| Yes | 5(29.4) | 16.0(1.5–169.5) | **0.02** | | **0.004** | 15.7(0.6–384.6) | 0.09 |
| No | 12(70.6) | – |  | |  |  |  |
| **TSC Mutation** |  |  |  | |  |  |  |
| *TSC2* mutated | 4(23.5) | 0.8(0.07–10.1) | 0.9 | | 0.99 |  |  |
| *TSC1*/*TSC2* Wild Type | 9(52.9) | 0.8 (0.09–8.1) | 0.9 | |  |  |  |
| *TSC1* mutated | 4(23.5) | **–** | **–** | |  |  |  |
| ***TP53*** |  |  |  | |  |  |  |
| Mutated | 5(29.4) | 2.0(0.4–9.5) | 0.4 | | 0.36 |  |  |
| Wild Type | 12(70.6) | **–** | **–** | |  |  |  |
| **Lines of therapy** |  |  |  | |  |  |  |
| 1 | 6(35.3) | **–** | **–** | |  | **–** | **–** |
| 2 | 5(29.4) | 16.9(1.0–272.0) | **0.04** | | **0.01** | 8.8(0.5–159.9) | 0.1 |
| $\geq$3 | 6(35.3) | 1.5(0.1–17.1) | 0.7 | |  | 0.4(0.007–20.9) | 0.6 |
| **TFE3** |  |  |  | |  |  |  |
| Negative | 11(64.7) | **–** | **–** | |  | **–** | **–** |
| Positive | 6(35.3) | 4.4(0.9–21.3) | 0.06 | | **0.04** | 24.9(1.2–522.9) | **0.03** |
| **History of Tuberous Sclerosis** |  |  |  | |  |  |  |
| Yes | 2(11.8) | 0.6(0.06–5.2) | 0.6 | | 0.62 |  |  |
| No | 15(88.2) | **–** | **–** | |  |  |  |
| **Treatment (First Line)** |  |  |  | |  |  |  |
| mTOR Inhibitors | 12(70.6) | 6.5(0.7–62.0) | 0.1 | | 0.06 | 3.9(0.04–354.6) | 0.5 |
| Chemotherapy | 5(29.4) | **–** | **–** | |  | **–** | **–** |
