# Supplementary material for: Systemic Treatments and Molecular Biomarkers for Perivascular Epithelioid Cell Tumors: A Single-institution Retrospective Analysis
Source: Cancer Res Commun. 2023 Jul 12;3(7):1212–23. doi: 10.1158/2767-9764.CRC-23-0139 (PMC10335919; doi:10.1158/2767-9764.CRC-23-0139)
Supplement: Table S8 — shows median clinical PFS from first-line therapy in months, as well as 5-year clinical PFS rate from first-line therapy for the whole cohort. [file crc-23-0139-s18.docx]

**Table S8A.** Median clinical PFS from first-line treatment for the whole cohort.

|  | **Patients  (*N*)** | **Events (*N*)** | **Median (months)** | **95% CI (months)** |
| --- | --- | --- | --- | --- |
| TFE3 positive | 6 | 3 | 121.5 | (6.0–NR) |
| TFE3 negative | 23 | 8 | 92.4 | (14.1–NR) |
| *TP53*_WT | 24 | 7 | 92.4 | (15.8–NR) |
| *TP53*_MUT | 5 | 4 | 9.4 | (8.9–NR) |
| *TSC1*/*TSC2*_WT | 20 | 6 | 92.4 | (15.8–NR) |
| *TSC1*_MUT | 4 | 2 | 9.4 | (6.0–NR) |
| *TSC2*_MUT | 5 | 3 | 9.3 | (8.9–NR) |
| Uterine | 9 | 6 | 9.4 | (9.3–NR) |
| Extra-uterine | 20 | 5 | 92.4 | (92.4–NR) |
| Malignant PEComa | 17 | 10 | 14.1 | (8.9–NR) |
| LAM/AML/Epithelioid AML | 12 | 1 | NR | (NA) |
| mTOR inhibitors | 24 | 8 | NR | (9.4–NR) |
| Chemotherapy | 5 | 3 | 92.4 | (15.8–NR) |
| Metastatic at diagnosis | 7 | 4 | 9.4 | (6.0–NR) |
| Localized at diagnosis | 22 | 7 | 92.4 | (15.8–NR) |

**Table S8B.** 5-year clinical PFS rate from first-line treatment for the whole cohort.

|  | ***N* at risk** | **Events (*N*)** | **5-year PFS rate (%)** | **95% CI  (%)** |
| --- | --- | --- | --- | --- |
| TFE3 positive | 4 | 1 | 62.5 | (32.0–100.0) |
| TFE3 negative | 11 | 1 | 60.6 | (41.5–88.4) |
| *TP53*_WT | 11 | 1 | 66.3 | (47.1–93.1) |
| *TP53*_MUT | 2 | 1 | 26.7 | (5.0–100.0) |
| *TSC1*/*TSC2*_WT | 11 | 1 | 69.3 | (49.8–96.3) |
| *TSC1*_MUT | 1 | 1 | 0 | (NA) |
| *TSC2*_MUT | 2 | 1 | 33.3 | (6.7–100.0) |
| Uterine | 3 | 1 | 30.5 | (9.8–95.2) |
| Extra-uterine | 10 | 1 | 72.3 | (52.4–99.9) |
| Malignant PEComa | 4 | 1 | 31.6 | (12.9–77.2) |
| LAM/AML/Epithelioid AML | 10 | 1 | 90.0 | (73.2–100.0) |
| mTOR inhibitors | 10 | 1 | 58.4 | (39.7–86.0) |
| Chemotherapy | 3 | 1 | 66.7 | (22.9–100.0) |
| Metastatic at diagnosis | 4 | 1 | 42.9 | (18.2–100.0) |
| Localized at diagnosis | 10 | 1 | 66.0 | (445.6–95.6) |

NA: not available; NR: not reached; PEComa: perivascular epithelioid cell tumors; LAM: lymphangioleiomyomatosis; AML: angiomyolipoma; LAM: lymphangioleiomyomatosis; ICI: immune checkpoint inhibitors; WT: wild-type; MUT: mutated; mTOR: mammalian target of rapamycin.
