# Supplementary material for: Systemic Treatments and Molecular Biomarkers for Perivascular Epithelioid Cell Tumors: A Single-institution Retrospective Analysis
Source: Cancer Res Commun. 2023 Jul 12;3(7):1212–23. doi: 10.1158/2767-9764.CRC-23-0139 (PMC10335919; doi:10.1158/2767-9764.CRC-23-0139)
Supplement: Table S9 — shows Cox proportional hazard analysis results for clinical progression-free survival from first-line therapy in patients with malignant PEComa. [file crc-23-0139-s19.docx]

**Table S9**. Cox proportional hazard analysis for clinical progression-free survival from first-line therapy in patients with malignant PEComa.

| **Variables** | ***N* Episodes (%)** | **Univariable** | | | | **Multivariable** | |
| --- | --- | --- | --- | --- | --- | --- | --- |
|  |  | **Hazard Ratio**  **(95%CI)** | ***P*-value**  **(Cox-Wald)** | ***P*-value**  **(Log-Rank)** | | **Hazard Ratio**  **(95%CI)** | ***P*-value**  **(Cox-Wald)** |
| **Sex** |  |  |  | |  |  |  |
| Male | 4(23.5) | 0.6(0.1–2.9) | 0.5 | | 0.50 |  |  |
| Female | 13(76.5) | **–** | **–** | |  |  |  |
| **Primary Site** |  |  |  | |  |  |  |
| Uterine | 9(52.9) | 0.8(0.2–3.2) | 0.8 | | 0.80 |  |  |
| Extra-uterine | 8(47.1) | **–** | **–** | |  |  |  |
| **Age, years (median, range)** | 48(5–71) | 1.1(1.0–1.2) | 0.01 | | **0.003** | 1.1(1.02–1.2) | **0.01** |
| **Metastatic at diagnosis** |  |  |  | |  |  |  |
| Yes | 5(29.4) | 5.4(0.9–29.6) | 0.05 | | **0.03** | 7.8(0.2–241.8) | 0.2 |
| No | 12(70.6) |  |  | |  |  |  |
| **TSC Mutation** |  |  |  | |  |  |  |
| *TSC2* mutated | 4(23.5) | 0.5(0.07–3.4) | 0.5 | | 0.53 |  |  |
| *TSC1*/*TSC2* Wild Type | 9(52.9) | 0.4 (0.06–2.2) | 0.3 | |  |  |  |
| *TSC1* mutated | 4(23.5) | **–** | **–** | |  |  |  |
| ***TP53*** |  |  |  | |  |  |  |
| Mutated | 5(29.4) | 1.5(0.4–5.4) | 0.5 | | 0.53 |  |  |
| Wild Type | 12(70.6) | **–** | **–** | |  |  |  |
| **Lines of therapy** |  |  |  | |  |  |  |
| 1 | 6(35.3) | **–** | **–** | |  | **–** | **–** |
| 2 | 5(29.4) | 8.0(1.1–56.7) | **0.03** | | **0.03** | 0.7 (0.03–18.5) | 0.8 |
| $\geq$3 | 6(35.3) | 1.1(0.2–5.9) | 0.8 | |  | 0.04(0.00004–39.5) | 0.4 |
| **TFE3** |  |  |  | |  |  |  |
| Negative | 11(64.7) | **–** | **–** | |  |  |  |
| Positive | 6(35.3) | 1.1(0.3–4.2) | 0.9 | | 0.93 |  |  |
| **History of Tuberous Sclerosis** |  |  |  | |  |  |  |
| Yes | 2(11.8) | 0.6(0.1–3.2) | 0.6 | | 0.62 |  |  |
| No | 15(88.2) | **–** | **–** | |  |  |  |
| **Treatment (First Line)** |  |  |  | |  |  |  |
| mTOR Inhibitors | 12(70.6) | 2.0(0.5–8.5) | 0.3 | | 0.33 | 0.3(0.01–7140.3) | 0.5 |
| Chemotherapy | 5(29.4) | **–** | **–** | |  | **–** | **–** |
| **Adjuvant Treatment** |  |  |  | |  |  |  |
| Yes |  | 0.7(0.2–2.8) | 0.7 | | 0.6 | 9.9(0.003–6920.9) | 0.5 |
| No |  |  |  | |  |  |  |
