# Supplementary material for: Systemic Treatments and Molecular Biomarkers for Perivascular Epithelioid Cell Tumors: A Single-institution Retrospective Analysis
Source: Cancer Res Commun. 2023 Jul 12;3(7):1212–23. doi: 10.1158/2767-9764.CRC-23-0139 (PMC10335919; doi:10.1158/2767-9764.CRC-23-0139)
Supplement: Table S10 — shows median clinical PFS from first-line therapy in months, as well as 5-year clinical PFS rate from first-line therapy in patients with malignant PEComa. [file crc-23-0139-s20.docx]

**Table S10A.** Median clinical PFS from first-line treatment in patients

with malignant PEComa.

|  | **Patients  (*N*)** | **Events (*N*)** | **Median (months)** | **95% CI (months)** |
| --- | --- | --- | --- | --- |
| TFE3 positive | 11 | 7 | 14.1 | (9.3–NR) |
| TFE3 negative | 6 | 3 | 121.5 | (6.0–NR) |
| *TP53*_WT | 12 | 6 | 15.8 | (9.3–NR) |
| *TP53*_MUT | 5 | 4 | 9.4 | (8.9–NR) |
| *TSC1*/*TSC2*_WT | 9 | 5 | 15.8 | (7.6–NR) |
| *TSC1*_MUT | 4 | 2 | 9.4 | (6.0–NR) |
| *TSC2*_MUT | 4 | 3 | 9.3 | (8.9–NR) |
| Uterine | 9 | 6 | 9.4 | (9.3–NR) |
| Extra-uterine | 8 | 4 | 14.1 | (8.9–NR) |
| mTOR inhibitors | 12 | 7 | 9.3 | (7.6–NR) |
| Chemotherapy | 5 | 3 | 92.4 | (15.8–NR) |
| Metastatic at diagnosis | 5 | 4 | 7.6 | (6.0–NR) |
| Localized at diagnosis | 12 | 6 | 15.8 | (9.3–NR) |

**Table S10B.** 5-year clinical PFS rate from first-line treatment in patients

with malignant PEComa.

|  | ***N* at risk** | **Events (*N*)** | **5-year OS**  **rate (%)** | **95% CI  (%)** |
| --- | --- | --- | --- | --- |
| TFE3 positive | 4 | 1 | 62.5 | (32.0–100.0) |
| TFE3 negative | 3 | 1 | 25.4 | (7.7–83.8) |
| *TP53*_WT | 3 | 1 | 33.4 | (11.3–99.3) |
| *TP53*_MUT | 2 | 1 | 26.7 | (5.1–100.0) |
| *TSC1*/*TSC2*_WT | 3 | 1 | 37.0 | (12.7–100.0) |
| *TSC1*_MUT | 1 | 1 | 0 | NA |
| *TSC2*_MUT | 2 | 1 | 33.3 | (6.7–100.0) |
| Uterine | 3 | 1 | 30.5 | (9.7–95.2) |
| Extra-uterine | 2 | 1 | 30.0 | (6.3–100.0) |
| mTOR inhibitors | 2 | 1 | 18.0 | (3.5–93.4) |
| Chemotherapy | 3 | 1 | 66.7 | (29.9–100.0) |
| Metastatic at diagnosis | 2 | 1 | 20.0 | (3.5–100.0) |
| Localized at diagnosis | 4 | 1 | 42.9 | (18.2–100.0) |

NR: not reached; WT: wild-type; MUT: mutated; mTOR: mammalian target of rapamycin
