# Supplementary material for: Systemic Treatments and Molecular Biomarkers for Perivascular Epithelioid Cell Tumors: A Single-institution Retrospective Analysis
Source: Cancer Res Commun. 2023 Jul 12;3(7):1212–23. doi: 10.1158/2767-9764.CRC-23-0139 (PMC10335919; doi:10.1158/2767-9764.CRC-23-0139)
Supplement: Table S11 — shows median clinical PFS for all treatment episodes in months, as well as 5-year clinical PFS rate for all treatment episodes in the whole cohort. [file crc-23-0139-s21.docx]

**Table S11A.** Median clinical PFS for all treatment episodes in the whole cohort.

|  | **Patients  (*N*)** | **Events (*N*)** | **Median (months)** | **95% CI (months)** |
| --- | --- | --- | --- | --- |
| TFE3 positive | 13 | 8 | 12.5 | (2.3–NR) |
| TFE3 negative | 46 | 21 | 18.4 | (13.0–NR) |
| *TP53*_WT | 49 | 22 | 18.4 | (13.1–NR) |
| *TP53*_MUT | 10 | 7 | 8.9 | (6.0–NR) |
| *TSC1*/*TSC2*_WT | 38 | 18 | 18.43 | (14.14–NR) |
| *TSC1*_MUT | 11 | 5 | 9.43 | (6.0–NR) |
| *TSC2*_MUT | 10 | 6 | 9.29 | (8.86–NR) |
| Uterine | 25 | 16 | 13.0 | (9.3–NR) |
| Extra-uterine | 34 | 13 | 34.8 | (14.1–NR) |
| Malignant PEComa | 42 | 25 | 13.0 | (9.3–20.7) |
| LAM/AML/Epithelioid AML | 17 | 3 | NR | (NA) |
| mTOR inhibitors | 40 | 20 | 18.4 | (9.4–NR) |
| Chemotherapy | 9 | 5 | 15.8 | (12.5–NR) |
| ICI | 5 | 1 | NR | (NA) |
| Other*^a^* | 5 | 3 | 13.1 | (13.1–NR) |
| Metastatic at diagnosis | 13 | 7 | 9.4 | (6.0–NR) |
| Localized at diagnosis | 46 | 22 | 17.4 | (13–NR) |

**Table S11B.** 5-year clinical PFS for all treatment episodes in the whole cohort.

|  | ***N* at risk** | **Events (*N*)** | **5-year PFS rate (%)** | **95% CI  (%)** |
| --- | --- | --- | --- | --- |
| TFE3 positive | 2 | 1 | 19.0 | (3.5–100.0) |
| TFE3 negative | 10 | 1 | 40.0 | (25.8–61.9) |
| *TP53*_WT | 10 | 1 | 40.0 | (25.9–61.7) |
| *TP53*_MUT | 2 | 1 | 16.4 | (2.8–96.0) |
| *TSC1*/*TSC2*_WT | 8 | 1 | 39.7 | (24.3–64.7) |
| *TSC1*_MUT | 2 | 1 | 20.2 | (3.7–100.0) |
| *TSC2*_MUT | 3 | 1 | 31.1 | (10.1–96.2) |
| Uterine | 5 | 1 | 22.8 | (9.7–53.4) |
| Extra-uterine | 7 | 1 | 46.2 | (28.3–75.5) |
| Malignant PEComa | 6 | 1 | 19.5 | (9.0–42.3) |
| LAM/AML/Epithelioid AML | 10 | 1 | 76.6 | (56.5–100.0) |
| mTOR inhibitors | 9 | 1 | 38.5 | (24.2–61.2) |
| Chemotherapy | 3 | 1 | 40.0 | (13.7–100.0) |
| ICI | 5 | 1 | 80.0 | (51.6–100.0) |
| Other | 1 | 1 | 0.0 | (NA) |
| Metastatic at diagnosis | 3 | 1 | 29.3 | (10.1–85.3) |
| Localized at diagnosis | 9 | 1 | 38.3 | (24.0–61.0) |

NR: not reached; NA: not available; PEComa: perivascular epithelioid cell tumors; LAM: lymphangioleiomyomatosis; AML: angiomyolipoma; LAM: lymphangioleiomyomatosis; ICI: immune checkpoint inhibitors; WT: wild-type; MUT: mutated; mTOR: mammalian target of rapamycin; Other*^a^*: olaparib (*n*=1), pazopanib (*n*=1), pazopanib-everolimus (*n*=1), anastrozole (*n*=1), and levantinib-everolimus (*n*=1).
