# Supplementary material for: Systemic Treatments and Molecular Biomarkers for Perivascular Epithelioid Cell Tumors: A Single-institution Retrospective Analysis
Source: Cancer Res Commun. 2023 Jul 12;3(7):1212–23. doi: 10.1158/2767-9764.CRC-23-0139 (PMC10335919; doi:10.1158/2767-9764.CRC-23-0139)
Supplement: Table S12 — shows results of Cox proportional hazard analysis for progression-free survival for all treatment episodes combined, regardless of line of therapy in patients with malignant PEComa only. [file crc-23-0139-s22.docx]

**Table S12**. Cox proportional hazard analysis for progression-free survival for all treatment episodes combined, regardless of line of therapy in malignant PEComa only

| **Variables** | ***N* Episodes (%)** | | **Univariable** | | | |  |  |
| --- | --- | --- | --- | --- | --- | --- | --- | --- |
|  | |  | **Hazard Ratio**  **(95%CI)** | ***P*-value**  **(Cox-Wald)** | ***P*-value**  **(Log-Rank)** | |  |  |
| **Sex** | |  |  |  | |  |  |  |
| Male | | 7(16.7) | 0.7(0.2–2.3) | 0.6 | | 0.5 |  |  |
| Female | | 35(83.3) | **–** | **–** | |  |  |  |
| **Primary Site** | |  |  |  | |  |  |  |
| Uterine | | 25(59.5) | 0.8(0.4–1.9) | 0.7 | | 0.7 |  |  |
| Extra-uterine | | 17(40.5) | **–** | **–** | |  |  |  |
| **Age, years (median, range)** | | 50(5–71) | 1.0(0.9–1.0) | 0.1 | | 0.1 |  |  |
| **Metastatic at diagnosis** | |  |  |  | |  |  |  |
| Yes | | 11(26.2) | 2.3(0.9–5.8) | 0.07 | | 0.06 |  |  |
| No | | 31(73.8) | – |  | |  |  |  |
| **TSC Mutation** | |  |  |  | |  |  |  |
| *TSC2* mutated | | 9(21.5) | 0.8(0.2–2.8) | 0.8 | | 0.9 |  |  |
| *TSC1*/*TSC2* Wild Type | | 22(52.3) | 0.9(0.3–2.6) | 0.9 | |  |  |  |
| *TSC1* mutated | | 11(26.2) | **–** | **–** | |  |  |  |
| ***TP53*** | |  |  |  | |  |  |  |
| Mutated | | 10(23.8) | 1.3(0.6–3.3) | 0.5 | | 0.5 |  |  |
| Wild Type | | 32(76.2) | **–** | **–** | |  |  |  |
| **Lines of therapy** | |  |  |  | |  |  |  |
| 1 | | 6(14.3) | **–** | **–** | |  |  |  |
| 2 | | 10(23.8) | 4.0(0.9–17.1) | 0.06 | | 0.1 |  |  |
| $\geq$3 | | 26(61.9) | 2.1(0.6–7.7) | 0.2 | |  |  |  |
| **TFE3** | |  |  |  | |  |  |  |
| Negative | | 29(69.0) | **–** | **–** | |  |  |  |
| Positive | | 13(31.0) | 1.4(0.6–3.4) | 0.4 | | 0.4 |  |  |
| **Frailty (Patient ID)** | | **–** | **–** | **–** | |  |  |  |
| **History of Tuberous Sclerosis** | |  |  |  | |  |  |  |
| Yes | | 4(9.5) | 0.8(0.3–2.5) | 0.8 | | 0.8 |  |  |
| No | | 38(90.5) | **–** | **–** | |  |  |  |
| **Treatment (All Lines)** | |  |  |  | |  |  |  |
| mTOR Inhibitors | | 26(61.9) | 1.5(0.5–4.1) | 0.4 | | 0.7 | 1.2(0.4–3.4) | 0.7 |
| Other*^a^* | | 4(9.5) | 2.5(0.5–13.7) | 0.3 | |  | 1.9(0.3-10.5) | 0.5 |
| ICI | | 3(7.1) | 2.8(0.3–26.9) | 0.4 | |  | 2.1(0.2-20.6) | 0.5 |
| Chemotherapy | | 9(21.4) | **–** | **–** | |  |  |  |
| **Adjuvant treatment** | |  |  |  | |  |  |  |
| Yes | | 7(16.7) | 0.4(0.1–1.3) | 0.1 | | 0.1 | 0.5(0.2–1.5) | 0.2 |
| No | | 35(83.3) |  |  | |  |  |  |
